# Associations of dairy, meat, and fish intakes with risk of incident dementia and with cognitive performance: the Kuopio Ischaemic Heart Disease Risk Factor Study (KIHD)

**DOI:** 10.1007/s00394-022-02834-x

**Published:** 2022-02-25

**Authors:** Maija P. T. Ylilauri, Sari Hantunen, Eija Lönnroos, Jukka T. Salonen, Tomi-Pekka Tuomainen, Jyrki K. Virtanen

**Affiliations:** 1 Institute of Public Health and Clinical Nutrition, University of Eastern Finland, P.O. Box 1627, 70211 Kuopio, Finland; 2 MAS-Metabolic Analytical Services Oy, Helsinki, Finland; 3 Department of Public Health, The Faculty of Medicine, The University of Helsinki, Helsinki, Finland

**Keywords:** Apolipoprotein E4, Cognitive performance, Dairy, Dementia, Fish, Meat

## Abstract

**Purpose:**

To investigate if dairy, meat, and fish intakes associate with dementia and cognitive performance.

**Methods:**

We included 2497 dementia-free men from Eastern Finland, aged 42–60 years in 1984–1989 at the baseline examinations. Data on cognitive tests [Mini Mental State Exam (MMSE), trail making test (TMT), verbal fluency test (VFL), selective reminding test (SRT), and Russell’s adaptation of the visual reproduction test (VRT)] at the 4-year re-examinations were available for 482 men and on the *ApoE* phenotype for 1259 men. Data on dementia events were obtained by linkage to national health registers. Diet was assessed with baseline 4-day food records. Cox regression and analysis of covariance were used for analyses.

**Results:**

During a mean 22-year follow-up, 337 men had a dementia diagnosis. Among the foods, only cheese intake associated with dementia risk (hazard ratio in the highest vs. the lowest quartile = 0.72, 95% confidence interval = 0.52–0.99, *P*-trend = 0.05). In the cognitive tests, higher non-fermented dairy and milk intakes associated with worse verbal fluency (VFT). Higher processed red meat intake associated with worse verbal (SRT) and visual memory (VRT), whereas higher unprocessed red meat intake associated with better general cognitive functioning (MMSE) and processing speed and executive functioning (TMT). Higher fish intake associated with better verbal memory (SRT). Among *APOE*-ε*4* carriers, especially non-fermented dairy intake associated with higher risk of dementia outcomes, and higher fish intake indicated better cognitive performance.

**Conclusion:**

Although higher intake of some food groups associated with cognitive performance, we found little evidence for associations with dementia risk.

**Supplementary Information:**

The online version contains supplementary material available at 10.1007/s00394-022-02834-x.

## Introduction

Alzheimer’s Disease International has estimated in 2019 that over 50 million people worldwide are suffering from dementia, causing yearly about one trillion-dollar global costs [1]. By 2050, the number of people having dementia is likely to rise to over 150 million [1]. As cure for vascular and neurodegenerative diseases causing dementia does not yet exist, prevention or onset-delay are ways to lower the individual and social burden of the condition. One of the midlife modifiable factors for dementia may be diet [2]. A healthy diet has had a protective association with the risk of dementia in several studies [2]. Dietary factors also contribute to the risk of obesity, type 2 diabetes, hypercholesterolemia, and hypertension, which may in turn increase the risk of dementia [2].

Although the potential role of diet in dementia prevention is recognized, it is still unclear, which particular food items may have an association with the risk of cognitive decline. In our previous study, we found a trend toward a lower risk of dementia with higher egg intake among Finnish men [3]. Higher egg intake was also associated with better performance in certain cognitive tests. Similar associations with egg intake have also been observed in other studies [4, 5].

When it comes to other animal products, the associations have been inconsistent. Evidences concerning the associations between dairy intake and dementia or cognitive performance have been too heterogeneous to draw firm conclusions of their relations [6, 7]. Also, the evidence regarding the association between specific meat sources and cognitive function or dementia outcomes is limited and inconclusive [8]. Moreover, the studied sources have basically been types of meat, such as beef, pork, or lamb [8], and studies that are investigating processed vs. unprocessed red meat are called for, as these meat subtypes may have a different health impact [9]. In contrast, evidence concerning the associations between higher fish consumption and lower risk of dementia [10, 11] or Alzheimer’s disease (AD) [10–12] has been more constant, although the association with all-cause dementia has not always been observed [12]. There is also indication of the association between higher fish consumption and better performance in cognitive tests [13]; however, associations may depend on the types of fish consumed [14].

In this study, we examined the associations of dairy, meat, and fish intakes with incident dementia in 2497 men from Eastern Finland without diagnosed cognitive or memory disorders at the baseline. In a subset of 482 men, we investigated the associations of dairy, meat, and fish intakes with cognitive performance 4 years after the baseline examinations. The intakes of dairy and meat were also divided into subgroups. Moreover, we analyzed possible effect modification by the *APOE*-ε*4* phenotype in all of the associations, because the *APOE*-ε*4* phenotype is the major genetic risk factor for AD [15] and its prevalence is high in the Finnish population [16].

## Materials and methods

### Study population

**Fig. 1:**
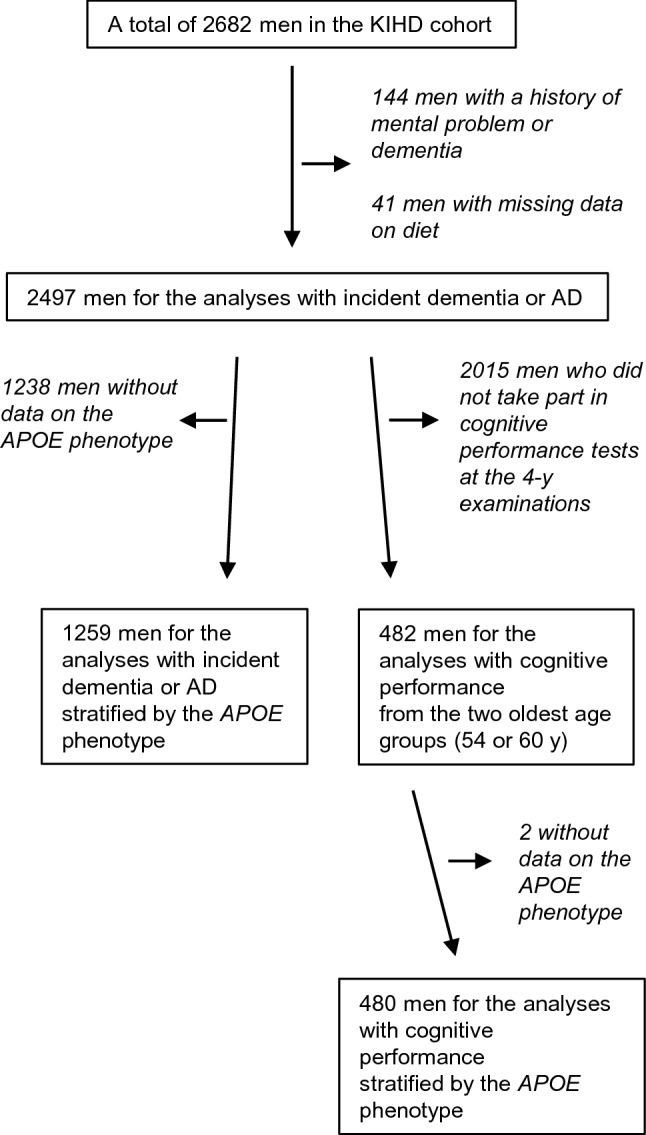
Number of subjects in the analyses. *AD* Alzheimer disease, *APOE* apolipoprotein E, *KIHD* Kuopio Ischaemic Heart Disease Risk Factor Study

The Kuopio Ischaemic Heart Disease Risk Factor Study (KIHD) was designed to primarily investigate risk factors for cardiovascular diseases, atherosclerosis, and related outcomes in a prospective, population-based sample of men from Eastern Finland [17]. Therefore, cognitive outcomes can be regarded as secondary outcomes of the KIHD study. The baseline examinations were carried out in 1984–1989. The study sample consisted of 3235 men living in Kuopio and surrounding areas who were 42, 48, 54, or 60 years old. Among those, 2682 (82.9%) participated in the baseline examinations in two cohorts (Supplemental Fig. 1). The first cohort consisted of 1166 men who were 54 years old, enrolled in 1984–1986, and the second cohort included 1516 men who were 42, 48, 54, or 60 years old, enrolled in 1986–1989. The baseline examinations were followed by the 4-year examination round in 1991–1993, in which 1038 men from the second cohort (88% of the eligible) participated. The baseline characteristics of the entire study population have been described [18]. The numbers of subjects in the analyses of incident dementia, AD, and cognitive performance and in the *APOE*-ε*4* stratified analyses are illustrated in Fig. 1.

### Assessment of dietary intakes

Baseline food consumption was assessed with guided food recording of 4 days, of which one was a weekend day, by household measures. A picture book of common foods and dishes was used to help in estimation of portion sizes. The picture book contained 126 most common foods and drinks consumed in Finland, and for each food item the participant could choose from 3 to 5 commonly used portion sizes or describe the portion size in relation to those in the book. To further improve accuracy, instructions were given and completed food records were checked by a nutritionist together with a participant. Food and nutrient intakes were estimated with the NUTRICA^®^ 2.5 software (Social Insurance Institution, Turku, Finland). All nutrients were energy-adjusted by the residual method [19]. The databank of the software is mainly based on Finnish values of nutrient composition of foods. The dairy food groups used in the calculations were total dairy, fermented dairy, non-fermented dairy, total milk, and cheese. The meat groups were total meat, red meat, processed red meat, and unprocessed red meat. The foods included in these variables are listed in Tables 1 and 2.

**Table 1:** Dairy products included in the dairy groups, their mean intakes ± SDs, (medians), and proportions

	g/day	% of intake
Total dairy	711 ± 360 (688)	100
Fermented dairy	189 ± 219 (106)	27
Non-fermented dairy	522 ± 331 (471)	73
Fermented dairy	189 ± 219 (106)	100
Low-fat fermented dairy (< 3.9% fat)^a^	165 ± 219 (84)	87
High-fat fermented dairy (≥ 3.9% fat)^b^	3 ± 16 (0)	2
Cheese	21 ± 25 (14)	11
Non-fermented dairy	522 ± 331 (471)	100
Low-fat non-fermented dairy (< 3.9% fat)^c^	306 ± 287 (217)	59
High-fat non-fermented dairy (≥ 3.9% fat)^d^	216 ± 304 (64)	41
Total milk	500 ± 327 (449)	100
Low-fat milk (< 3.5% fat)	306 ± 286 (216)	61
High-fat milk (≥ 3.5% fat)	194 ± 302 (25)	39
Cheese	21 ± 25 (14)	100
Low-fat cheese (< 17% fat)^e^	2 ± 9 (0)	10
High-fat cheese (≥ 17% fat)^f^	19 ± 23 (11)	90

^a^Includes sour milk, buttermilk, kefir, yogurt, curdled milk, and quark

^b^Includes sour cream, curdled milk, yogurt, and crème fraiche

^c^Includes low-fat milk (< 3.5%)

^d^Includes high-fat milk (≥ 3.5%), cream, ice cream, pudding, and Finnish squeaky cheese

^e^Includes medium-hard cheese, hard cheese, cottage cheese, cheese spread, egg cheese, and buttermilk cheese

^f^Includes medium–hard cheese, hard cheese, cheese spread, white mold cheese, blue cheese, and brined cheese

**Table 2:** Meat products included in the meat groups, their mean intakes ± SDs, (medians), and proportions

	g/day	% of intake
Total meat	159 ± 80 (149)	100
Red meat	144 ± 77 (134)	91
Unprocessed white meat^a^	10 ± 28 (0)	6
Offal	5 ± 13 (0)	3
Red meat	144 ± 77 (134)	100
Unprocessed red meat^b^	69 ± 48 (62)	48
Processed red meat^c^	70 ± 60 (58)	49
Game^d^	5 ± 21 (0)	3

^a^Includes chicken and turkey. There were no users of processed white meat

^b^Includes pork, beef, and lamb

^c^Includes marinated meat, bacon, canned meat, jellied meat, cold cuts, and sausages made of red meat

^d^Includes horsemeat, reindeer, venison, hare meat, and willow grouse meat

Fish was calculated as a single variable as we were not able to divide fish intake into subgroups by fish species or by processing style. In the baseline characteristics tables, the egg consumption variable represents total egg consumption, including eggs in mixed dishes and recipes. The intake of choline refers to a sum of free choline, glycerophosphocholine, phosphocholine, phosphatidylcholine and sphingomyelin. Because no information on choline and phosphatidylcholine values in Finnish foods exist, the values for these nutrients in the diet are based on the USDA database [20].

### Measurements

Venous blood samples were collected between 8 and 10AM at the baseline examinations. Subjects were instructed to abstain from ingesting alcohol for 3 days and from smoking and eating for 12 h prior to giving the sample. Detailed descriptions of the determination of serum lipids and lipoproteins, assessment of medical history and medications at baseline, family history of diseases, smoking, alcohol intake, blood pressure, and physical activity have been published [21, 22]. Serum high-sensitivity C-reactive protein (CRP) was measured with an immunometric assay (Immulite High Sensitivity CRP Assay, DPC, Los Angeles, CA, USA). Education was assessed in years by self-administered questionnaire. Body mass index was computed as the ratio of weight in kilograms to the square of height in meters. The *APOE* phenotype was determined from blood samples of 1033 men who participated in the 4-year examinations and from 307 other men from the baseline examinations, about whom blood samples for phenotyping were available. The phenotype was determined from plasma with isoelectric focusing and immunoblotting techniques. Subjects who had the phenotype 3/4 or 4/4 were included in the *APOE-ε4* group. We have previously shown that those with the *APOE* 3/4 or 4/4 phenotype had 97% higher risk of dementia and 127% higher risk of AD in this study population, compared to the other phenotypes [3].

### Outcomes

Data on incident dementia or AD events from the beginning of the study to the end of the year 2014 were obtained by computer linkage to the national health registers [3]. International Classification of Diseases 8 (ICD-8) code 290, ICD-9 codes 4378A and 290, and ICD-10 codes F00, F01, F02, F03, G30, and G31 were included in dementia. ICD-8 codes 29000 and 29010, ICD-9 codes 290 and 3310A, and ICD-10 codes F00 and G30 were included in AD.

Cognitive performance was measured from 482 men at the 4-year examinations with the use of five neuropsychological tests: the Mini Mental State Exam, the trail making test A, the verbal fluency test, the selective reminding test, and the Russell’s adaptation of the visual reproduction test [3, 23–27]. The tests were administered by interviewers trained in neuropsychological assessment. Each of the tests has been validated in the Finnish population [28].

### Statistical analysis

The baseline characteristics among the whole study population were assessed by means. The univariate relationships between total dairy, total meat, and fish intakes and baseline characteristics were assessed by means and linear regression for continuous variables or *χ*^2^-tests for bivariate relationships. Cox proportional hazards regression models were used to estimate hazard ratios (HR) for incident dementia and AD. Schoenfeld residuals did not indicate significant evidence of violation of the proportional hazards assumption. The associations with cognitive tests were analysed with analysis of covariance (ANCOVA). Absolute risk reduction was calculated by multiplying the absolute risk in the reference group by the multivariable-adjusted HR reduction in the comparison group. The associations with the risk of incident dementia or AD were evaluated in quartiles of dairy, meat, and fish intakes. However, the associations with cognitive tests were evaluated in tertiles due to the limited number of participants in the subset. The associations with the risk of incident dementia or AD and with the cognitive tests were also analysed continuously per each 50 g/day higher intakes.

The confounders in the analyses were selected based on the confounders used in our previous studies [3, 29], established risk factors for dementia, previously published associations with dementia [30], or on associations with exposures or outcomes in the present analysis. The Model 1 included age (years), baseline examination year, and energy intake (kcal/day). The multivariable model (Model 2) included the model 1 and education years, pack-years of smoking (cigarette packs/day × years of smoking), body mass index (kg/m^2^), diabetes (yes/no), leisure-time physical activity (kcal/day), history of coronary heart disease (yes/no), use of lipid-lowering medication (yes/no), intakes of alcohol (g/week), fiber (g/day), sum of fruits, berries and vegetables (g/day), and dietary fat quality (ratio of PUFA plus MUFA to SFA plus trans fatty acids).

Cohort mean was used to replace missing values in continuous covariates [31] (< 2.4% of values). There were no missing values in categorical variables. Statistical significance of the interactions on a multiplicative scale was assessed by stratified analysis and likelihood ratio tests by a cross-product term. Tests of linear trend were conducted by assigning the median values for each category of exposure variable and treating those as a single continuous variable*.* All *P* values were 2-tailed (*α* = 0.05). Data were analysed by SPSS 25 for Windows (Armonk, NY: IBM Corp.).

## Results

### Baseline characteristics

Mean ± SD (median) energy-adjusted total dairy intake was 711 ± 360 (688) g/day, total meat intake 159 ± 80 (149) g/day, and fish intake 46 ± 54 (31) g/day. The detailed information on dairy and meat intakes is shown in Tables 1 and 2. The other baseline characteristics of the men in the whole study population are described in Table 3.

**Table 3:** Baseline characteristics of the 2497 men from the Kuopio Ischaemic Heart Disease Risk Factor Study

Age (years)	53 ± 5.1 (54.3)
Education (years)	8.6 ± 3.4 (8.0)
Marital status, married (%)	87
Annual income (euro)	13,406 ± 8987 (11,864)
Body mass index (kg/m^2^)	26.9 ± 3.6 (26.4)
Leisure-time physical activity (kcal/day)	141 ± 175 (84)
Current smoker (%)	29
Hypertension (%)	60
Coronary heart disease (%)	25
Stroke (%)	3
Diabetes (%)	6
Lipid lowering medication at baseline (%)	0.6
Lipid lowering medication during follow-up (%)	48
Systolic blood pressure, mmHg	134 ± 17 (132)
Diastolic blood pressure, mmHg	89 ± 10 (88)
Serum total cholesterol, mmol/L	5.91 ± 1.08 (5.84)
Serum LDL cholesterol, mmol/L	4.05 ± 1.01 (3.96)
Serum HDL cholesterol, mmol/L	1.29 ± 0.30 (1.26)
Serum triglycerides, mmol/L	1.31 ± 0.83 (1.11)
Serum long-chain omega-3 polyunsaturated fatty acids (%)^a^	4.7 ± 1.6 (4.3)
Blood glucose (mmol/L)	4.8 ± 1.2 (4.6)
Serum CRP (mg/L)	2.43 ± 4.15 (1.28)
Alcohol intake (g/wk)	74 ± 134 (31)
*Dietary intakes*	
Energy (kcal/day)	2440 ± 622 (2398)
Protein (*E*%)	15.8 ± 2.5 (15.5)
Fat (*E*%)	38.7 ± 5.9 (38.6)
Saturated fatty acids (*E*%)	18.2 ± 4.1 (18.0)
Polyunsaturated fatty acids (*E*%)	4.5 ± 1.4 (4.3)
Monounsaturated fatty acids (*E*%)	11.7 ± 2.2 (11.6)
Trans fatty acids (*E*%)	1.1 ± 0.4 (1.0)
Cholesterol (mg/day)	401 ± 107 (388)
Carbohydrates (*E*%)	42.7 ± 6.5 (42.8)
Fiber (g/day)	25.1 ± 7.1 (24.4)
Choline (mg/day)	431 ± 88 (424)
Phosphatidylcholine (mg/day)	188 ± 63 (180)
Eggs (g/day)	32 ± 25 (26)
Fish (g/day)	46 ± 54 (31)
Grains (g/day)	254 ± 92 (242)
Whole grains (g/day)	159 ± 75 (149)
Fruits, berries, and vegetables (g/day)	250 ± 155 (226)
Potatoes (g/day)	162 ± 88 (150)
Fat spreads and oils (g/day)	56 ± 24 (53)
Butter and butter containing spreads (g/day)	36 ± 28 (32)
Vegetable margarines (g/day)	18 ± 17 (12)
Vegetable oils (g/day)	2 ± 4 (1)
Tea (mL/day)	94 ± 173 (0)
Coffee (mL/day)	564 ± 292 (563)

Values are means ± SD or percentages (medians in parentheses)

^a^Sum of serum eicosapentaenoic acid, docosapentaenoic acid and docosahexaenoic acid concentrations, indicated as proportion of all serum fatty acids

The baseline characteristics according to availability of apolipoprotein E phenotype and cognitive test data are described in Supplemental Tables 1 and 2, respectively. The participants whose *APOE* phenotype data were available had a slightly more optimal health and dietary markers compared to the participants whose *APOE* phenotype data were not available. However, those participants with the known phenotype had somewhat less favourable lipid profile (Supplemental Table 1). The subpopulation of those who completed the cognitive tests did not remarkably differ from the rest of the study population. However, the prevalence of coronary heart disease was higher among the subpopulation, but this may be explained by the higher age among them (Supplemental Table 1).

Men with a higher total dairy intake had, in general, unhealthier lifestyle habits than the men with a lower dairy intake. They were older, less educated, less likely married, had lower income, were less physically active during the free time, were more likely to smoke, and had a lower serum long-chain omega-3 PUFA concentration. Their energy, protein, fat, SFA, cholesterol, and choline intakes were higher. In contrast, their MUFA, total PUFA, fiber, phosphatidylcholine, and alcohol intakes were lower compared with the men with a lower dairy intake (Supplemental Table 3).

Men with a higher total meat intake were younger, had higher income, higher BMI, and were more likely smokers and were more likely to have type 2 diabetes. However, they had less likely hypertension and a history of coronary heart disease. They also had higher energy, protein, fat, SFA, MUFA, total PUFA, cholesterol, choline, phosphatidylcholine, and alcohol intakes. Their carbohydrate and fiber intakes were lower compared with the men with a lower meat intake (Supplemental Table 4).

Men with a higher fish intake were older, had a higher BMI, were more likely smokers and had a history of coronary heart disease. Their serum total and LDL cholesterol concentrations and long-chain omega-3 PUFA concentration were higher. Instead, their serum triglycerides concentration was lower. The intakes of energy, protein, total PUFA, cholesterol, choline, and alcohol were higher compared with the men with a lower fish intake. Instead, the intakes of fat, SFA, MUFA, and carbohydrates were lower (Supplemental Table 5).

### Dairy intake and risk of dementia and AD

During the mean ± SD follow-up of 21.9 ± 7.9 years (range 0.02–30.8 years), 337 men (13.5%) were diagnosed with dementia and 266 (10.7%) with AD. Total dairy, fermented dairy, non-fermented dairy, and total milk intakes were not associated with the risk of incident dementia (Table 4) or AD (Supplemental Table 6). However, those in the highest (> 31 g/day) compared with the lowest (< 0.7 g/day) cheese intake quartile had 28% (95% CI: 1%, 48%; *P*-trend across quartiles = 0.05) lower multivariable-adjusted risk of incident dementia (absolute risk in the lowest quartile = 31.8%; absolute risk reduction in the highest quartile = 9.0%). When evaluated continuously, each 50 g/day higher cheese intake was associated with 20% lower multivariable-adjusted risk of incident dementia, although the association was not statistically significant (*P* = 0.10). Similar point estimates were observed between cheese intake and risk of incident AD, although the associations were not statistically significant either in quartiles or in continuous evaluation (Supplemental Table 6).

**Table 4:** Risk of dementia in quartiles of dairy, meat, and fish intakes among 2497 men from the Kuopio Ischaemic Heart Disease Risk Factor Study

	Intake quartile				*P-*trend	Per 50 g/day increase	*P* value
	1	2	3	4			
Total dairy							
Intake, g/day (median)	< 455 (292)	455–687 (580)	688–927 (802)	> 927 (1119)			
*N* of events/participants	68/624 (10.9%)	88/624 (14.1%)	90/625 (14.4%)	91/624 (14.6%)			
Model 1^a^	1	1.20 (0.87, 1.65)^b^	1.12 (0.81, 1.55)	1.14 (0.80, 1.62)	0.58	1.01 (0.99, 1.03)	0.21
Model 2^c^	1	1.28 (0.92, 1.76)	1.15 (0.82, 1.60)	1.27 (0.87, 1.84)	0.31	1.02 (1.00, 1.04)	0.07
Fermented dairy							
Intake, g/day (median)	< 24 (3)	24–106 (56)	107–285 (184)	> 285 (443)			
*N* of events/participants	93/623 (14.9%)	76/623 (12.2%)	84/627 (13.4%)	84/624 (13.5%)			
Model 1^a^	1	0.75 (0.56, 1.02)	0.80 (0.59, 1.07)	0.77 (0.57, 1.04)	0.26	1.00 (0.98, 1.02)	0.97
Model 2^c^	1	0.76 (0.56, 1.03)	0.83 (0.62, 1.13)	0.82 (0.61, 1.11)	0.51	1.00 (0.98, 1.03)	0.78
Non-fermented dairy							
Intake, g/day (median)	< 265 (158)	265–471 (372)	472–728 (585)	> 728 (904)			
*N* of events/participants	72/624 (11.5%)	79/624 (12.7%)	102/625 (16.3%)	84/624 (13.5%)			
Model 1^a^	1	1.06 (0.77, 1.46)	1.30 (0.95, 1.77)	1.11 (0.78, 1.57)	0.40	1.01 (0.99, 1.03)	0.18
Model 2^c^	1	1.10 (0.80, 1.53)	1.39 (1.02, 1.90)	1.13 (0.79, 1.63)	0.34	1.02 (1.00, 1.04)	0.10
Total milk							
Intake, g/day (median)	< 244 (144)	244–449 (351)	450–705 (564)	> 705 (875)			
*N* of events/participants	75/624 (12.0%)	76/624 (12.2%)	101/625 (16.2%)	85/624 (13.6%)			
Model 1^a^	1	0.96 (0.70, 1.32)	1.25 (0.92, 1.69)	1.12 (0.79, 1.57)	0.29	1.01 (0.99, 1.03)	0.18
Model 2^c^	1	1.00 (0.73, 1.39)	1.33 (0.97, 1.81)	1.14 (0.80, 1.62)	0.26	1.02 (1.00, 1.04)	0.11
Cheese							
Intake, g/day (median)	< 0.7 (0)	0.7–14 (8)	15–31 (21)	> 31 (49)			
*N* of events/participants	107/709 (15.1%)	81/544 (14.9%)	79/620 (12.7%)	70/624 (11.2%)			
Model 1^a^	1	0.83 (0.62, 1.11)	0.67 (0.50, 0.90)	0.74 (0.55, 1.00)	0.05	0.80 (0.62, 1.03)	0.08
Model 2^c^	1	0.83 (0.62, 1.11)	0.71 (0.53, 0.96)	0.72 (0.52, 0.99)	0.05	0.80 (0.62, 1.04)	0.10
Total meat							
Intake, g/day (median)	< 106 (77)	106–151 (128)	152–204 (174)	> 204 (261)			
*N* of events/participants	89/624 (14.3%)	88/625 (14.1%)	86/623 (13.8%)	74/625 (11.8%)			
Model 1^a^	1	1.04 (0.77, 1.40)	1.09 (0.80, 1.47)	1.09 (0.78, 1.52)	0.58	1.03 (0.96, 1.11)	0.40
Model 2^c^	1	1.02 (0.75, 1.38)	1.03 (0.76, 1.41)	1.01 (0.70, 1.44)	0.96	1.01 (0.93, 1.10)	0.80
Red meat							
Intake, g/day (median)	< 91(65)	91–134 (113)	135–187 (156)	> 187 (230)			
*N* of events/participants	91/624 (14.6%)	88/625 (14.1%)	85/624 (13.6%)	73/624 (11.7%)			
Model 1^a^	1	0.99 (0.74, 1.33)	1.02 (0.76, 1.38)	1.01 (0.72, 1.40)	0.93	1.03 (0.95, 1.11)	0.53
Model 2^c^	1	0.98 (0.73, 1.32)	0.97 (0.71, 1.31)	0.93 (0.65, 1.31)	0.66	1.00 (0.92, 1.09)	1.00
Processed red meat							
Intake, g/day (median)	< 25 (10)	25–57 (40)	58–97 (76)	> 97 (139)			
*N* of events/participants	85/637 (13.3%)	91/608 (15.0%)	83/626 (13.3%)	78/626 (12.5%)			
Model 1^a^	1	1.08 (0.80, 1.45)	1.13 (0.83, 1.53)	1.20 (0.87, 1.66)	0.25	1.06 (0.97, 1.17)	0.19
Model 2^c^	1	1.06 (0.79, 1.44)	1.11 (0.81, 1.51)	1.12 (0.79, 1.57)	0.53	1.04 (0.94, 1.15)	0.50
Unprocessed red meat							
Intake, g/day (median)	< 39 (21)	39–67 (53)	68–103 (81)	> 103 (132)			
*N* of events/participants	90/624 (14.4%)	91/624 (14.6%)	79/625 (12.6%)	77/624 (12.3%)			
Model 1^a^	1	0.93 (0.70, 1.25)	0.87 (0.64, 1.18)	0.84 (0.61, 1.15)	0.27	0.96 (0.86, 1.08)	0.52
Model 2^c^	1	0.95 (0.71, 1.28)	0.87 (0.64, 1.18)	0.83 (0.60, 1.14)	0.21	0.96 (0.85, 1.08)	0.45
Fish							
Intake, g/day (median)	< 3 (0)	3–31 (18)	32–66 (48)	> 66 (102)			
*N* of events/participants	78/623 (12.5%)	94/625 (15.0%)	72/624 (11.5%)	93/625 (14.9%)			
Model 1^a^	1	1.10 (0.82, 1.49)	0.81 (0.59, 1.12)	1.14 (0.84, 1.54)	0.61	1.00 (0.91, 1.11)	0.92
Model 2^c^	1	1.12 (0.83, 1.52)	0.82 (0.59, 1.13)	1.08 (0.79, 1.47)	0.95	0.99 (0.89, 1.10)	0.85

^a^Model 1 adjusted for age, baseline examination year, and energy intake

^b^Values are hazard ratios (95% confidence intervals)

^c^Model 2 adjusted for the Model 1 and education years, pack-years of smoking (cigarette packs/day × years of smoking), body mass index (kg/m^2^), diabetes (yes/no), leisure-time physical activity (kcal/day), history of coronary heart disease (yes/no), use of lipid-lowering medication (yes/no), intakes of alcohol (g/week), fiber (g/day), sum of fruits, berries and vegetables (g/day), and dietary fat quality (ratio of polyunsaturated fatty acids plus monounsaturated fatty acids to saturated fatty acids plus trans fatty acids)

In the subset of 1259 men, 33% had the *APOE-ε4* phenotype (Supplemental Table 7). Each 50 g/day higher total dairy intake was associated with 5% (95% CI 1%, 8%) higher multivariable-adjusted risk of incident dementia among the *APOE-ε4* carriers (*P*-interaction 0.03). No evidence was found for effect modification by the *APOE-ε4* phenotype with intakes of fermented dairy, non-fermented dairy, total milk, or cheese (*P*-interactions > 0.07, Supplemental Fig. 2).

Among the *APOE-ε4* carriers, each 50 g/day higher total dairy intake was associated with 6% (95% CI: 2%, 10%) higher multivariable-adjusted risk of incident AD (*P*-interaction 0.007), each 50 g/day higher non-fermented dairy intake with 5% (95% CI 1%, 9%) higher risk (*P*-interaction 0.03), and each 50 g/day higher total milk intake with 5% (95% CI 1%, 9%) higher risk (*P*-interaction 0.03). No evidence was found for effect modification by the *APOE-ε4* phenotype with intakes of fermented dairy or cheese and AD (*P*-interactions > 0.19, Supplemental Fig. 3).

### Meat intake and risk of dementia and AD

The intakes of total meat, red meat, processed red meat, or unprocessed red meat were not associated with the risk of dementia (Table 4) or AD (Supplemental Table 6). The *APOE-ε4* phenotype did not modify any of the associations, either (Supplemental Figs. 2 and 3).

### Fish intake and risk of dementia and AD

The intake of fish was not associated with the risk of dementia (Table 4) or AD (Supplemental Table 6) after multivariable adjustments and the *APOE-ε4* phenotype did not modify the associations (Supplemental Figs. 2 and 3).

### Complete case and sensitivity analyses with dementia and AD

We performed complete case analyses (*n* = 2416) to investigate the impact of replacing missing values in covariates. The associations in these analyses were generally similar as in the original analyses (Supplemental Table 8 and 9).

Within 11 years, which was half of the mean follow-up period of 22 years that was used in the main analyses, only 16 dementia cases occurred. Hence, sensitivity analyses with a shorter follow-up time could not be done.

### Dairy intake and cognitive performance

In the subset of 482 men, higher non-fermented dairy and total milk intakes were associated with worse performance in the Verbal Fluency Test at the 4-year examinations after multivariable adjustments (Supplemental Fig. 4). Men in the highest non-fermented dairy intake tertile produced 3.0 words less compared with men in the lowest tertile (95% CI − 5.7, − 0.3 words; *P*-trend = 0.03), and men in the highest total milk intake tertile produced 2.9 words less compared to those in the lowest tertile (95% CI − 5.6, − 0.2 words; *P*-trend = 0.03).

In the analyses of 480 men stratified by the *APOE-ε4* phenotype, no evidence for effect modification with groups of dairy products and any of the cognitive tests was found after multivariable adjustments (Supplemental Figs. 5–9).

### Meat intake and cognitive performance

Higher intake of processed red meat was associated with worse performance in the selective reminding test (Supplemental Fig. 10) and in the Russell’s adaptation of the visual reproduction test (Supplemental Fig. 11) at the 4-year examinations after multivariable adjustments. Compared with men in the lowest tertile, men in the highest tertile of processed red meat intake recalled 2.5 words less in the selective reminding test (95% CI − 4.4, − 0.7 words; *P*-trend = 0.008) and scored 1.0 points less in the visual reproduction test (95% CI − 1.8, − 0.2 words; *P*-trend = 0.01).

In contrast, higher intake of unprocessed red meat was associated with better performance in the Mini Mental State Exam (Supplemental Fig. 12) and in the trail making test A (Supplemental Fig. 13). Men in the highest unprocessed red meat tertile scored 0.4 points more in the Mini Mental State Exam compared with men in the lowest tertile (95% CI − 0.02, 0.9 points; *P*-trend = 0.06). Those in the highest unprocessed red meat intake tertile also had 4.9 s faster performance in the trail making test A compared with those in the lowest tertile (95% CI − 8.8, − 1.0 words; *P*-trend = 0.01).

No evidence for effect modification by the *APOE-ε4* phenotype between meat intake and any of the cognitive tests was found after multivariable adjustments (Supplemental Figs. 5–9).

### Fish intake and cognitive performance

Higher intake of fish was associated with a better performance in the Selective Reminding Test at the 4-year re-examinations (Supplemental Fig. 10). The men in the highest fish intake tertile scored 1.9 points more (95% CI 0.1, 3.7; *P*-trend = 0.04) compared to the men in the lowest tertile.

In the analyses stratified by the *APOE-ε4* phenotype, higher fish intake was associated with a better performance in the verbal fluency test among the *APOE-ε4* carriers but not among the non-carriers after multivariable adjustments (*P*-interaction = 0.03). The *APOE-ε4* carriers in the highest fish intake tertile produced 5.8 more words compared with those in the lowest tertile (95% CI 0.8, 10.8 words; *P*-trend = 0.02). However, when evaluated continuously, no statistically significant effect modification was observed (*P*-interaction = 0.24, Supplemental Fig. 5). No evidence was found for the effect modification by the *APOE-ε4* phenotype with the other tests (Supplemental Figs. 6–9).

## Discussion

In this population-based cohort study, higher cheese intake associated with lower risk of incident dementia, whereas other dairy or meat subgroups or fish did not associate with the risk of incident dementia, and none of the foods associated with AD risk. Higher intakes of non-fermented dairy, total milk, and processed red meat associated with a worse performance in at least one cognitive test, whereas higher intakes of unprocessed red meat and fish associated with a better performance. The *APOE-ε4* phenotype modified some associations with dairy and fish intakes.

To our best knowledge, this is the first prospective cohort study to investigate the association between cheese intake and risk of developing dementia. In a recent case–control study by Filippini et al. [32], associations between cheese intake and dementia outcomes were not found. Nonetheless, a beneficial association between cheese intake and cognitive performance is supported in the majority of previous studies [33–36]. For potential explanations for the better cognitive performance, the probiotic effect of lactid acid bacteria through the gut-brain axis [36], high level of vitamin K2 [36], and role of bioactive compounds [34] and the amino acid tyramine [33] that are high in cheese have been discussed.

In general, the evidence between dairy intake and dementia outcomes or cognitive performance is incoherent [6, 7, 37, 38] and may be product specific [33–36, 39]. Two systematic reviews have suggested that higher dairy intake may have a beneficial association with cognitive performance [37, 38], but it may be limited to Asian populations [38]. For example, in the Japanese population, the average daily dairy intake was 85 g [40], when in our study it was more than eightfold, 711 g. Hence, in populations with traditionally low dairy intake, increase in the intake may reduce dementia risk [40]. Instead, in countries with high dairy intake, such as in Finland, the plateau may have already been reached [36] with no further health benefits [7]. Nevertheless, associations of some specific non-fermented dairy products, such as whole fat dairy products and dairy desserts, with worse cognitive performance have been reported [41, 42]. These findings for non-fermented dairy intake are in line with our results. Overall, further studies are needed to determine the optimal amount and type of dairy products for brain health.

The evidence concerning meat consumption and dementia outcomes or cognitive performance is inconclusive. According to the systematic review by Zhang et al. [8], most of the studies did not find an association between meat intake and cognitive outcomes, although a meta-analysis of five studies by the same authors showed a protective associations between higher meat intake and cognitive disorders [8]. However, the associations may be different if processed and lean meat are studied separately [9]. Consumption of unprocessed, lean meat may be favorable [43] and processed meat unfavorable [44], but findings are inconsistent [45]. Similar to our associations with cognitive performance, in a recent UK Biobank study higher processed meat intake associated with higher risk and higher unprocessed meat intake with lower risk of dementia and AD, without effect modification by the APOE phenotype [46]. The role of inflammation in red meat consumption [47] and dietary nitrite [45] in processed red meat may explain the adverse health effects. Cultural differences in the types of meats consumed and the cooking methods may also explain the incoherent study results [45]. It is likely that meat cannot be studied as a single category, as pork, beef, lamb, game, poultry, as well as their processed forms may have different impacts on health.

Compared to dairy and meat, association between fish intake and dementia outcomes or cognitive performance is more coherent. According to systematic reviews and meta-analyses, higher fish consumption associates with lower risk of dementia or AD [10–12, 48–50]. The association between fish intake and cognitive performance is not as clear, as only one [13] of the two published systematic reviews [12, 13] has found a beneficial association. However, in most of the original studies the follow-up time may have been too short or the number of participants too low for statistically significant associations. This is supported by Samieri et al. [13]. They pooled cohorts of five studies with null findings. In their meta-analysis of 23,688 participants in total, however, higher fish intake was associated with better cognitive performance, which is in line with our finding. It is also noteworthy that the approximately 22-year follow-up time in our study was much longer than the follow-up time (range 3.9–9.1 years) in any of the original studies in the pooled analysis by Samieri et al. [13].

In dementia prevention, reducing cardiovascular diseases may be one approach, e.g. via the cardioprotective effect of the long-chain omega-3 PUFAs in fish [11, 12]. Indeed, especially higher intake of fatty fish has been associated with lower risk of dementia or AD [51] or with better verbal memory [14], whereas non-significant associations have been found with other fish types [14, 51]. We did not have information on fish species, but we have previously reported that higher concentrations of serum long-chain omega-3 PUFAs, mainly a marker of fatty fish intake in the KIHD, were associated with better cognitive performance [52]. Although we saw an association between higher fish intake and better cognitive performance in verbal memory, other associations were not evident. Hence, 4-day food recording may not accurately assess intake of foods that are commonly consumed 1–2 times/week, such as fish, whereas serum long-chain omega-3 PUFAs are an objective biomarker for intake of few weeks [53]. Also, not all fish are high in omega-3 fatty acids, which may partly explain some null findings with fish. Fish may also have independent health benefits on brain, which may not be explained by the omega-3 PUFAs [54, 55].

In general, the *APOE-ε4* phenotype did not modify most associations, and due to many tested associations, some of the observed interactions may be incidental findings. The findings suggest that higher intake of total dairy, and especially non-fermented dairy and total milk, may have a more adverse impact among the *APOE-ε4* carriers, whereas higher intake of fish may be more favorable for the carries vs. non-carriers. However, as few studies have investigated the impact of the *APOE* phenotype on the associations between diet and cognitive decline and the findings are inconsistent [10, 13, 51, 55, 56], future studies are needed to elucidate the role of the *APOE* phenotype in the diet-dementia relationship.

Our study has several strengths: population-based recruitment, detailed information about diet and potential confounders, investigation of meat and dairy intakes in subgroups, long follow-up, register-based information on incident cases of dementia with no loss to follow-up, and information on the *APOE* phenotype. It is also an advantage that multiple cognitive tests were used, as many other studies only rely on a single test, such as Mini Mental State Exam, which may not be sensitive enough to detect sub-clinical decline. Using a set of cognitive tests facilitates the detection of subtle changes or changes that may only occur in a single cognitive domain. Hence, the null findings in some previous studies may partly result from unsensitive or limited use of cognitive test methods.

Potential limitations also exist: dietary data were collected only at baseline, which may have attenuated the associations with incident dementia. Information on fish species was not available. We did not have data on cognitive performance at the baseline, although we excluded participants with known mental problem including dementia. Data on tests and information on the *APOE*-ε*4* phenotype were available only for part of the participants, which limited the power to find associations. Our results may not be generalizable to persons diagnosed with cognitive or memory disorders, to women, or to ethnically diverse populations other than Caucasian.

In conclusion, our results may imply that higher intakes of non-fermented dairy, total milk, and processed red meat may have an adverse association with cognitive performance, whereas higher intake of unprocessed red meat and fish may have a favorable association. However, this influence may not mediate the risk of incident dementia or AD risk, as we found little evidence for associations with the risk of incident dementia, apart from the potential inverse association between cheese intake and dementia risk. In general, the *APOE*-ε*4* phenotype did not modify most of the associations. For verifying these tentative results, more studies are needed that investigate the association of different types of protein sources with dementia incidence or cognitive performance and consider the *APOE*-ε*4* phenotype. In addition, more uniform test batteries for cognitive performance assessment are needed, as the use of test methods in the current literature is heterogenous, making it difficult to draw conclusions.

## Supplementary Information

Below is the link to the electronic supplementary material.

Supplementary file1 (PDF 1360 kb)

## Data Availability

Data described in this manuscript will not be made available, because they contain sensitive personal data of the subjects, which cannot be completely anonymized.

## Abbreviations

AD: Alzheimer’s disease
*APOE-**ε**4*: Apolipoprotein E ε4
CRP: C-reactive protein
ICD: International classification of diseases
KIHD: Kuopio Ischaemic Heart Disease Risk Factor Study
PUFA: Polyunsaturated fatty acid
