# Supplementary material for: Associations of dairy, meat, and fish intakes with risk of incident dementia and with cognitive performance: the Kuopio Ischaemic Heart Disease Risk Factor Study (KIHD)
Source: Eur J Nutr. 2022 Feb 25;61(5):2531–42. doi: 10.1007/s00394-022-02834-x (PMC9279192; doi:10.1007/s00394-022-02834-x)
Supplement: Supplementary file 1 — Supplementary file1 (PDF 1360 kb) [file 394_2022_2834_MOESM1_ESM.pdf]

European Journal of Nutrition

Associations of dairy, meat, and fish intakes with risk of incident dementia and with cognitive performance: the Kuopio Ischaemic Heart Disease Risk Factor Study (KIHD)

Maija P.T. Ylilauri[1], Sari Hantunen[1], Eija Lönnroos[1], Jukka T. Salonen[2], Tomi-Pekka Tuomainen[1], Jyrki K. Virtanen[1]

[1]Institute of Public Health and Clinical Nutrition, University of Eastern Finland, P.O. Box 1627, 70211 Kuopio, Finland
[2]MAS-Metabolic Analytical Services Oy, Helsinki, Finland and the University of Helsinki, the Faculty of Medicine, Department of Public Health, Finland

Corresponding author: Jyrki K Virtanen, University of Eastern Finland, Institute of Public Health and Clinical Nutrition, Kuopio, Finland. Phone: +358-40-3552957, E-mail: jyrki.virtanen@uef.fi.

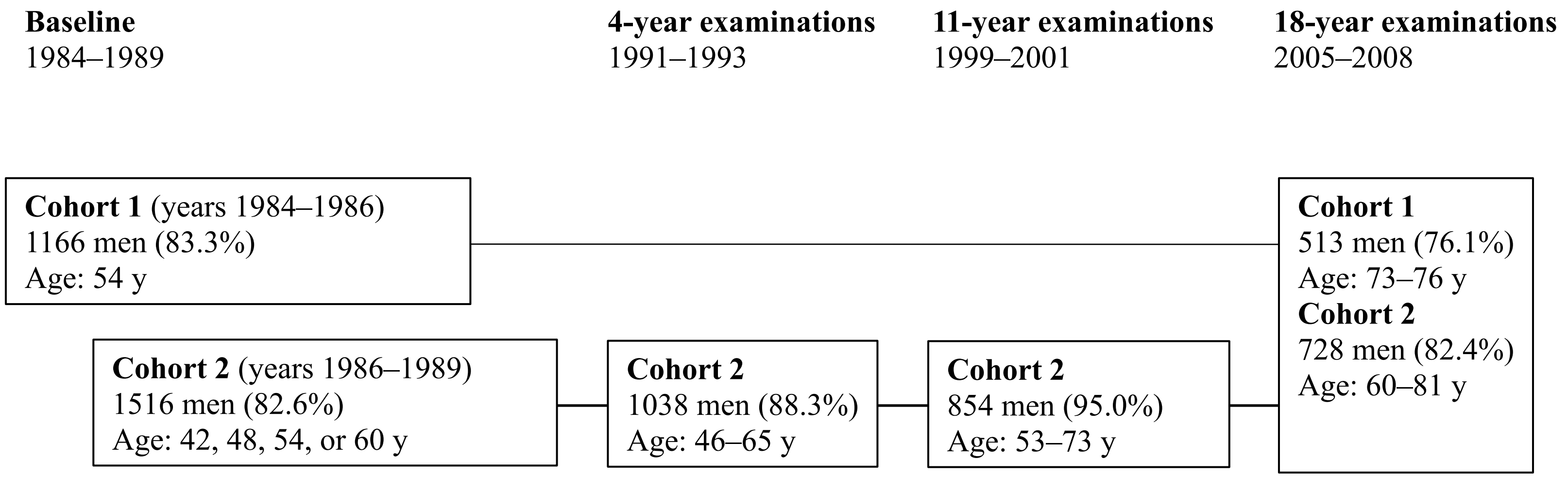

SUPPLEMENTAL FIGURE 1

Timeline of the Kuopio Ischaemic Heart Disease Risk Factor Study

The percentages in parentheses indicate the proportion of eligible participants that participated in the study visits.

SUPPLEMENTAL TABLE 1

Baseline characteristics according to availability of apolipoprotein E phenotype data among 2497 men from the Kuopio Ischaemic Heart Disease Risk Factor Study[1]

	Availability of apolipoprotein E phenotype data		
	Yes (n=1259)	No (n=1238)	*P*-difference
Age (years)	52.5±6.1 (54.3)	53.6±3.9 (54.3)	<0.001
Education (years)	9.0±3.5 (8.0)	8.3±3.4 (8.0)	<0.001
Marital status, married (%)	89	85	0.003
Annual income (euro)	14628±9315 (12881)	12167±8468 (10508)	<0.001
Body mass index (kg/m^2)	26.7±3.2 (26.4)	27.0±3.9 (26.5)	0.02
Leisure-time physical activity (kcal/d)	140±157 (88)	142±191 (78)	0.84
Current smoker (%)	27	31	0.10
Hypertension (%)	58	62	0.04
Coronary heart disease (%)	22	27	0.002
Stroke (%)	2	3	0.11
Diabetes (%)	4	8	<0.001
Lipid lowering medication at baseline (%)	1	0	0.05
Lipid lowering medication during follow-up (%)	54	41	<0.001
Systolic blood pressure, mmHg	133±16 (131)	136±18 (134)	<0.001
Diastolic blood pressure, mmHg	88±10 (88)	89±11 (89)	0.002
Serum total cholesterol, mmol/L	5.99±1.08 (5.93)	5.82±1.07 (5.67)	<0.001
Serum LDL cholesterol, mmol/L	4.11±1.02 (4.09)	3.98±1.00 (3.86)	0.001
Serum HDL cholesterol, mmol/L	1.28±0.29 (1.24)	1.30±0.31 (1.26)	0.25
Serum triglycerides, mmol/L	1.39±0.82 (1.20)	1.23±0.83 (1.03)	<0.001
Serum long-chain omega-3 polyunsaturated fatty acids (%)[2]	4.6±1.6 (4.3)	4.7±1.6 (4.4)	0.16
Blood glucose (mmol/L)	4.7±0.9 (4.5)	4.9±1.4 (4.6)	<0.001
Serum CRP (mg/L)	2.26±4.17 (1.22)	2.59±4.12 (1.36)	0.05
Alcohol intake (g/wk)	71±114 (31)	77±152 (31)	0.24
Dietary intakes			
Energy (kcal/d)	2426±593 (2387)	2456±650 (2400)	0.23
Protein (E%)	15.9±2.6 (15.7)	15.6±2.5 (15.3)	0.001
Fat (E%)	38.2±6.0 (38.3)	39.2±5.8 (39.2)	<0.001
Saturated fatty acids (E%)	17.6±4.0 (17.3)	18.8±4.1 (18.6)	<0.001

Polyunsaturated fatty acids (E%)	4.7±1.4 (4.5)	4.3±1.4 (4.1)	<0.001
Monounsaturated fatty acids (E%)	11.8±2.3 (11.6)	11.7±2.2 (11.5)	0.30
Trans fatty acids (E%)	1.0±0.4 (1.0)	1.1±0.4 (1.0)	0.15
Cholesterol (mg/d)	390±105 (378)	412±108 (400)	<0.001
Carbohydrates (E%)	43.2±6.6 (43.2)	42.3±6.3 (42.3)	0.001
Fiber (g/d)	25.6±7.5 (25.0)	24.5±6.6 (23.9)	<0.001
Choline (mg/d)	429±85 (422)	434±91 (426)	0.23
Phosphatidylcholine (mg/d)	187±60 (179)	188±65 (181)	0.75
Total dairy (g/d)	675±346 (649)	747±370 (724)	<0.001
Fermented dairy (g/d)	180±207 (103)	198±231 (113)	0.05
Non-fermented dairy (g/d)	495±320 (453)	550±340 (498)	<0.001
Total milk (g/d)	472±317 (422)	528±336 (478)	<0.001
Cheese (g/d)	23±26 (16)	19±23 (10)	<0.001
Total meat (g/d)	163±83 (151)	156±77 (146)	0.04
Unprocessed red meat (g/d)	70±47 (64)	68±48 (60)	0.19
Processed red meat (g/d)	70±61 (56)	70±59 (60)	0.98
White meat (g/d)	12±30 (0)	9±26 (0)	0.008
Game (g/d)	6±22 (0)	5±19 (0)	0.10
Offal (g/d)	5±13 (0)	5±13 (0)	0.50
Eggs (g/d)	30±23 (25)	33±26 (27)	0.007
Fish (g/d)	46±54 (31)	46±54 (31)	0.91
Grains (g/d)	260±97 (245)	247±87 (239)	<0.001
Whole grains (g/d)	162±80 (151)	156±69 (148)	0.02
Fruits, berries, and vegetables (g/d)	257±155 (234)	243±154 (221)	0.03
Potatoes (g/d)	157±83 (147)	167±93 (153)	0.003
Fat spreads and oils (g/d)	54±24 (51)	57±24 (55)	0.004
Butter and butter containing spreads (g/d)	33±27 (29)	39±27 (36)	<0.001
Vegetable margarines (g/d)	19±17 (14)	16±16 (11)	<0.001
Vegetable oils (g/d)	2±4 (1)	2±3 (0)	0.02
Tea (mL/d)	94±173 (0)	94±173 (0)	0.95
Coffee (mL/d)	571±291 (563)	556±292 (550)	0.20

[1]Values are means±SD or percentages (medians in parentheses).

[2]Sum of serum eicosapentaenoic acid, docosapentaenoic acid and docosahexaenoic acid concentrations, indicated as proportion of all serum fatty acids.

SUPPLEMENTAL TABLE 2

Baseline characteristics according to availability of cognitive test data among 2497 men from the Kuopio Ischaemic Heart Disease Risk Factor Study[1]

	Availability of cognitive test data		
	Yes (n=482)	No (n=2015)	*P*-difference
Age (years)	57.6±3.0 (55.1)	52.0±4.9 (54.3)	<0.001
Education (years)	8.3±3.3 (8.0)	8.7±3.5 (8.0)	0.01
Marital status, married (%)	88	87	0.61
Annual income (euro)	13665±8252 (12034)	13344±9153 (11864)	0.49
Body mass index (kg/m^2)	27.0±3.4 (26.7)	26.8±3.6 (26.4)	0.22
Leisure-time physical activity (kcal/d)	138±148 (86)	142±181 (83)	0.65
Current smoker (%)	28	30	0.27
Hypertension (%)	63	59	0.12
Coronary heart disease (%)	30	23	0.002
Stroke (%)	3	2	0.60
Diabetes (%)	5	6	0.33
Lipid lowering medication at baseline (%)	1	1	0.22
Lipid lowering medication during follow-up (%)	52	50	0.04
Systolic blood pressure, mmHg	134±17 (133)	134±17 (132)	0.71
Diastolic blood pressure, mmHg	88±10 (87)	89±11 (88)	0.01
Serum total cholesterol, mmol/L	5.80±1.04 (5.76)	5.94±1.09 (5.85)	0.01
Serum LDL cholesterol, mmol/L	3.91±0.94 (3.86)	4.08±1.03 (3.99)	<0.001
Serum HDL cholesterol, mmol/L	1.29±0.31 (1.23)	1.29±0.30 (1.26)	0.88
Serum triglycerides, mmol/L	1.48±0.84 (1.27)	1.27±0.82 (1.09)	<0.001
Serum long-chain omega-3 polyunsaturated fatty acids (%)[2]	4.6±1.7 (4.3)	4.7±1.6 (4.4)	0.70
Blood glucose (mmol/L)	4.8±1.1 (4.6)	4.8±1.2 (4.6)	0.51
Serum CRP (mg/L)	2.32±3.00 (1.35)	2.45±4.39 (1.26)	0.53
Alcohol intake (g/wk)	67±116 (26)	75±138 (33)	0.23
Dietary intakes			
Energy (kcal/d)	2340±569 (2322)	2464±632 (2410)	<0.001
Protein (E%)	16.2±2.7 (15.9)	15.7±2.5 (15.4)	<0.001
Fat (E%)	37.2±6.4 (37.2)	39.0±5.8 (39.1)	<0.001
Saturated fatty acids (E%)	17.0±4.2 (16.6)	18.5±4.0 (18.3)	<0.001
Polyunsaturated fatty acids (E%)	4.6±1.4 (4.5)	4.5±1.4 (4.3)	0.13

Monounsaturated fatty acids (E%)	11.5±2.3 (11.4)	11.8±2.2 (11.6)	0.005
Trans fatty acids (E%)	1.1±0.4 (1.0)	1.1±0.4 (1.0)	0.61
Cholesterol (mg/d)	379±109 (370)	406±106 (394)	<0.001
Carbohydrates (E%)	44.0±6.9 (44.0)	42.4±6.3 (42.5)	<0.001
Fiber (g/d)	26.8±8.0 (26.1)	24.6±6.8 (24.0)	<0.001
Choline (mg/d)	429±81 (421)	432±90 (425)	0.47
Phosphatidylcholine (mg/d)	182±60 (174)	189±63 (181)	0.02
Total dairy (g/d)	685±336 (664)	717±365 (694)	0.06
Fermented dairy (g/d)	192±216 (116)	188±220 (104)	0.70
Non-fermented dairy (g/d)	492±310 (455)	529±336 (477)	0.02
Total milk (g/d)	471±307 (433)	507±332 (453)	0.02
Cheese (g/d)	21±25 (13)	21±25 (14)	0.96
Total meat (g/d)	150±78 (146)	162±81 (150)	0.005
Unprocessed red meat (g/d)	68±49 (62)	69±48 (62)	0.657
Processed red meat (g/d)	59±55 (45)	73±61 (61)	<0.001
White meat (g/d)	12±30 (0)	10±28 (0)	0.10
Game (g/d)	7±24 (0)	5±19 (0)	0.23
Offal (g/d)	4±13 (0)	5±13 (0)	0.32
Eggs (g/d)	28±23 (23)	32±25 (27)	0.001
Fish (g/d)	49±57 (37)	45±53 (31)	0.19
Grains (g/d)	263±96 (247)	251±91 (241)	0.01
Whole grains (g/d)	170±84 (157)	156±72 (147)	0.001
Fruits, berries, and vegetables (g/d)	255±158 (234)	249±154 (225)	0.47
Potatoes (g/d)	148±79 (137)	165±90 (153)	<0.001
Fat spreads and oils (g/d)	52±23 (50)	56±25 (54)	0.001
Butter and butter containing spreads (g/d)	31±27 (26)	37±28 (34)	<0.001
Vegetable margarines (g/d)	19±18 (13)	17±17 (12)	0.02
Vegetable oils (g/d)	2±4 (1)	2±4 (1)	0.56
Tea (mL/d)	90±179 (0)	95±172 (0)	0.53
Coffee (mL/d)	534±264 (531)	571±298 (563)	0.01

[1]Values are means±SD or percentages (medians in parentheses).

[2]Sum of serum eicosapentaenoic acid, docosapentaenoic acid and docosahexaenoic acid concentrations, indicated as proportion of all serum fatty acids.

SUPPLEMENTAL TABLE 3

Baseline characteristics according to total dairy intake among 2497 men from the Kuopio Ischaemic Heart Disease Risk Factor Study[1]

	Total dairy intake quartile g/d (median)			
	1 <455 (292)	2 455–687 (580)	3 688–927 (802)	4 >927 (1119)
Number of subjects	624	624	625	624
Age (years)	52.4±5.5 (54.3)	53.0±5.3 (54.3)	53.7±4.8 (54.3)	53.2±4.9 (54.3)*
Education (years)	9.8±3.9 (9.0)	8.9±3.6 (8.0)	8.1±3.0 (7.0)	7.7±2.7 (7.0)*
Marital status, married (%)	91	86	87	85*
Annual income (euro)	16677±11425 (14237)	13326±7677 (12203)	12067±7740 (10678)	11537±7608 (10169)*
Body mass index (kg/m^2)	26.9±3.6 (26.2)	26.8±3.5 (26.6)	26.9±3.5 (26.6)	26.8±3.7 (26.4)
Leisure-time physical activity (kcal/d)	157±162 (103)	152±188 (91)	131±157 (78)	125±188 (66)*
Current smoker (%)	24	30	31	34*
Hypertension (%)	59	63	62	57
Coronary heart disease (%)	22	26	26	25
Stroke (%)	3	3	2	3
Diabetes (%)	6	6	6	5
Lipid lowering medication at baseline (%)	0.6	1.0	0.8	0.2
Lipid lowering medication during follow-up (%)	49	48	48	46
Systolic blood pressure, mmHg	134±16 (132)	134±18 (131)	134±16 (133)	135±18 (132)
Diastolic blood pressure, mmHg	89±10 (88)	89±11 (88)	89±10 (89)	89±11 (88)
Serum total cholesterol, mmol/L	5.87±1.10 (5.80)	5.94±1.08 (5.86)	5.92±1.09 (5.83)	5.90±1.04 (5.87)
Serum LDL cholesterol, mmol/L	4.02±1.01 (3.96)	4.06±1.02 (3.94)	4.06±1.03 (3.95)	4.05±1.00 (3.97)
Serum HDL cholesterol, mmol/L	1.28±0.31 (1.23)	1.28±0.32 (1.23)	1.28±0.28 (1.25)	1.32±0.30 (1.30)*
Serum triglycerides, mmol/L	1.30±0.78 (1.13)	1.37±0.77 (1.16)	1.34±0.98 (1.12)	1.25±0.75 (1.07)
Serum long-chain omega-3 polyunsaturated fatty acids (%)[b]	4.9±1.7 (4.6)	4.7±1.6 (4.3)	4.6±1.6 (4.3)	4.5±1.4 (4.2)*
Blood glucose (mmol/L)	4.8±1.1 (4.6)	4.8±1.4 (4.5)	4.8±1.1 (4.5)	4.8±1.1 (4.6)
Serum CRP (mg/L)	2.40±4.02 (1.26)	2.38±4.66 (1.23)	2.22±3.34 (1.27)	2.69±4.46 (1.34)
Alcohol intake (g/wk)	85±126 (43)	74±107 (36)	75±186 (26)	62±99 (21)*
Dietary intakes				

Energy (kcal/d)	2096±534 (2046)	2271±498 (2231)	2500±528 (2445)	2895±617 (2817)*
Protein (E%)	15.5±2.9 (15.1)	15.8±2.6 (15.4)	15.8±2.4 (15.6)	16.0±2.3 (15.8)*
Fat (E%)	38.0±6.0 (38.1)	38.8±5.8 (38.7)	38.3±5.9 (38.4)	39.7±6.0 (39.8)*
Saturated fatty acids (E%)	16.6±3.5 (16.4)	17.9±3.9 (17.5)	18.3±4.0 (18.3)	19.9±4.2 (20.1)*
Polyunsaturated fatty acids (E%)	5.2±1.5 (5.0)	4.7±1.4 (4.6)	4.3±1.2 (4.1)	3.8±1.1 (3.7)*
Monounsaturated fatty acids (E%)	12.1±2.5 (12.1)	12.0±2.2 (11.8)	11.4±2.1 (11.2)	11.3±1.9 (11.2)*
Trans fatty acids (E%)	1.0±0.4 (1.0)	1.1±0.4 (1.0)	1.1±0.4 (1.0)	1.1±0.3 (1.0)
Cholesterol (mg/d)	399±94 (391)	398±104 (389)	397±112 (381)	411±116 (396)*
Carbohydrates (E%)	42.8±7.2 (43.1)	42.5±6.3 (42.1)	43.3±6.3 (43.5)	42.3±6.0 (42.3)
Fiber (g/d)	25.7±6.6 (25.2)	24.9±6.7 (24.1)	25.8±7.8 (25.0)	23.9±7.2 (23.3)*
Choline (mg/d)	404±76 (400)	423±83 (414)	436±87 (429)	463±95 (454)*
Phosphatidylcholine (mg/d)	198±57 (189)	189±59 (181)	186±65 (176)	179±68 (169)*
Total dairy (g/d)	283±112 (292)	575±67 (580)	802±71 (802)	1185±248 (1119)*
Fermented dairy (g/d)	85±90 (53)	149±140 (111)	212±200 (156)	309±312 (200)*
Non-fermented dairy (g/d)	198±114 (186)	425±150 (451)	590±206 (639)	875±344 (897)*
Total milk (g/d)	182±113 (172)	403±150 (418)	568±207 (610)	846±340 (866)*
Cheese (g/d)	26±27 (18)	21±25 (14)	18±21 (12)	19±25 (10)*
Total meat (g/d)	163±84 (154)	158±81 (146)	150±73 (144)	166±81 (152)
Unprocessed red meat (g/d)	71±50 (63)	66±47 (59)	66±47 (59)	73±48 (67)
Processed red meat (g/d)	69±63 (53)	71±62 (58)	64±55 (54)	75±60 (64)
White meat (g/d)	14±31 (0)	11±31 (0)	9±26 (0)	7±24 (0)*
Game (g/d)	5±19 (0)	5±21 (0)	5±21 (0)	6±21 (0)
Offal (g/d)	4±11 (0)	5±12 (0)	5±13 (0)	5±15 (0)
Eggs (g/d)	29±22 (23)	29±23 (23)	33±26 (27)	36±27 (30)*
Fish (g/d)	48±56 (31)	47±55 (34)	44±51 (31)	46±54 (30)
Grains (g/d)	230±84 (220)	236±84 (226)	267±98 (254)	281±93 (271)*
Whole grains (g/d)	141±68 (132)	146±68 (138)	171±81 (157)	178±75 (167)*
Fruits, berries, and vegetables (g/d)	264±167 (236)	250±153 (231)	249±153 (225)	237±144 (216)*
Potatoes (g/d)	144±81 (135)	157±79 (150)	159±82 (146)	187±103 (172)*
Fat spreads and oils (g/d)	47±22 (46)	53±22 (51)	57±23 (55)	66±26 (63)*
Butter and butter containing spreads (g/d)	26±23 (22)	31±25 (28)	37±27 (35)	48±30 (46)*
Vegetable margarines (g/d)	18±16 (14)	19±18 (13)	17±17 (11)	16±16 (10)*

Vegetable oils (g/d)	3±4 (1)	2±4 (1)	2±4 (0)	2±3 (0)*
Tea (mL/d)	127±183 (42)	90±164 (0)	83±165 (0)	77±175 (0)*
Coffee (mL/d)	504±272 (494)	552±261 (550)	564±281 (550)	635±333 (625)*

**P*-trend across quartiles ≤0.05. *P*-trend was assessed with linear regression (continuous variables) or χ^2 test (bivariate relationships).

[1]Values are means±SD or percentages (medians in parentheses).

[2]Sum of serum eicosapentaenoic acid, docosapentaenoic acid and docosahexaenoic acid concentrations, indicated as proportion of all serum fatty acids.

SUPPLEMENTAL TABLE 4

Baseline characteristics according to total meat intake among 2497 men from the Kuopio Ischaemic Heart Disease Risk Factor Study[1]

	Total meat intake quartile g/d (median)			
	1 <104 (76)	2 104–148 (126)	3 149–203 (172)	4 >203 (250)
Number of subjects	624	625	623	625
Age (years)	54.5±4.4 (54.4)	53.3±4.7 (54.3)	53.0±5.2 (54.3)	51.4±5.7 (54.3)*
Education (years)	8.5±3.7 (8.0)	8.6±3.6 (8.0)	8.7±3.3 (8.0)	8.7±3.2 (8.0)
Marital status, married (%)	86	87	88	87
Annual income (euro)	12328±8506 (10678)	13658±9587 (11864)	13299±8771 (11864)	14326±8949 (12542)*
Body mass index (kg/m^2)	26.8±3.6 (26.4)	26.7±3.3 (26.4)	26.7±3.4 (26.3)	27.2±3.9 (26.7)*
Leisure-time physical activity (kcal/d)	148±180 (87)	144±201 (82)	138±156 (89)	134±158 (77)
Current smoker (%)	28	29	29	33*
Hypertension (%)	64	62	57	58*
Coronary heart disease (%)	28	24	25	22*
Stroke (%)	3	2	2	3
Diabetes (%)	5	4	8	7*
Lipid lowering medication at baseline (%)	0.6	0.8	0.8	0.3
Lipid lowering medication during follow-up (%)	46	49	47	50
Systolic blood pressure, mmHg	135±18 (133)	135±17 (133)	133±17 (131)	134±16 (132)
Diastolic blood pressure, mmHg	89±11 (88)	89±10 (88)	88±10 (87)	89±11 (89)
Serum total cholesterol, mmol/L	5.86±1.10 (5.8)	5.93±1.03 (5.8)	5.95±1.14 (5.9)	5.89±1.03 (5.9)
Serum LDL cholesterol, mmol/L	4.03±1.05 (3.9)	4.09±0.99 (4.0)	4.06±1.04 (4.0)	4.01±0.97 (4.0)
Serum HDL cholesterol, mmol/L	1.27±0.29 (1.2)	1.29±0.31 (1.3)	1.30±0.31 (1.3)	1.31±0.30 (1.3)
Serum triglycerides, mmol/L	1.32±0.80 (1.13)	1.29±0.68 (1.12)	1.34±0.94 (1.12)	1.30±0.86 (1.08)
Serum long-chain omega-3 polyunsaturated fatty acids (%)[2]	4.7±1.7 (4.4)	4.6±1.6 (4.3)	4.7±1.6 (4.4)	4.6±1.4 (4.3)
Blood glucose (mmol/L)	4.8±1.4 (4.5)	4.7±0.9 (4.6)	4.8±1.3 (4.6)	4.8±1.1 (4.6)
Serum CRP (mg/L)	2.38±4.53 (1.24)	2.23±3.25 (1.24)	2.36±3.54 (1.18)	2.74±5.03 (1.49)
Alcohol intake (g/wk)	56±101 (16)	66±118 (27)	74±130 (36)	100±173 (47)*
Dietary intakes				

Energy (kcal/d)	2159±543 (2110)	2400±590 (2358)	2455±561 (2430)	2747±645 (2682)*
Protein (E%)	15.2±2.5 (14.9)	15.6±2.4 (15.2)	15.9±2.6 (15.6)	16.5±2.5 (16.4)*
Fat (E%)	36.5±6.0 (36.6)	38.1±5.5 (37.9)	39.3±5.8 (39.5)	40.9±5.5 (41.0)*
Saturated fatty acids (E%)	17.7±4.5 (17.5)	18.2±4.0 (17.9)	18.3±4.0 (18.3)	18.5±3.7 (18.3)*
Polyunsaturated fatty acids (E%)	4.1±1.4 (3.8)	4.3±1.4 (4.1)	4.6±1.3 (4.5)	5.0±1.4 (4.9)*
Monounsaturated fatty acids (E%)	10.4±1.8 (10.3)	11.2±1.8 (11.1)	12.0±2.0 (12.0)	13.2±2.2 (13.1)*
Trans fatty acids (E%)	1.1±0.4 (1.1)	1.1±0.4 (1.0)	1.0±0.4 (1.0)	1.0±0.4 (1.0)*
Cholesterol (mg/d)	381±99 (368)	395±104 (381)	402±102 (392)	426±118 (412)*
Carbohydrates (E%)	45.9±6.5 (46.0)	43.7±5.9 (43.9)	42.0±5.9 (42.0)	39.3±5.7 (39.3)*
Fiber (g/d)	26.4±6.5 (25.9)	25.8±6.9 (25.2)	25.0±7.5 (23.9)	23.0±7.1 (22.5)*
Choline (mg/d)	403±78 (395)	425±83 (416)	436±81 (431)	463±99 (457)*
Phosphatidylcholine (mg/d)	162±56 (151)	180±58 (172)	193±55 (188)	216±69 (209)*
Total dairy (g/d)	695±323 (682)	734±363 (705)	693±344 (684)	722±403 (671)
Fermented dairy (g/d)	181±206 (117)	194±210 (123)	185±219 (96)	195±241 (98)
Non-fermented dairy (g/d)	514±300 (481)	540±337 (492)	508±320 (468)	526±365 (452)
Total milk (g/d)	495±298 (449)	517±333 (469)	484±312 (453)	504±362 (420)
Cheese (g/d)	19±23 (11)	22±26 (13)	20±22 (13)	24±28 (16)*
Total meat (g/d)	70±24 (76)	127±13 (126)	174±16 (172)	267±61 (250)*
Unprocessed red meat (g/d)	38±24 (37)	61±32 (61)	78±41 (75)	99±62 (93)*
Processed red meat (g/d)	26±23 (22)	51±33 (50)	74±42 (73)	128±74 (125)*
White meat (g/d)	3±11 (0)	5±16 (0)	11±25 (0)	21±45 (0)*
Game (g/d)	1±6 (0)	4±15 (0)	5±19 (0)	11±32 (0)*
Offal (g/d)	3±8 (0)	5±12 (0)	5±13 (0)	7±16 (0)*
Eggs (g/d)	29±24 (23)	32±24 (27)	32±24 (27)	34±27 (26)*
Fish (g/d)	54±56 (41)	48±54 (33)	43±53 (28)	40±52 (24)*
Grains (g/d)	245±85 (237)	256±96 (241)	255±93 (242)	259±94 (249)*
Whole grains (g/d)	155±71 (146)	160±74 (150)	161±79 (152)	159±75 (148)
Fruits, berries, and vegetables (g/d)	239±160 (213)	262±156 (236)	240±138 (222)	260±163 (238)
Potatoes (g/d)	143±84 (134)	166±85 (156)	166±91 (153)	172±91 (162)*
Fat spreads and oils (g/d)	52±23 (50)	55±25 (52)	57±24 (54)	58±26 (55)*
Butter and butter containing spreads (g/d)	33±26 (30)	36±28 (32)	37±26 (35)	36±29 (33)
Vegetable margarines (g/d)	17±17 (12)	17±16 (12)	17±16 (12)	19±19 (13)*

Vegetable oils (g/d)	2±3 (0)	2±3 (0)	2±3 (1)	3±4 (2)*
Tea (mL/d)	94±170 (0)	87±165 (0)	92±165 (0)	104±191 (0)
Coffee (mL/d)	543±277 (531)	567±289 (563)	573±300 (563)	571±300 (563)

**P*-trend across quartiles ≤0.05. *P*-trend was assessed with linear regression (continuous variables) or χ^2 test (bivariate relationships).

[1]Values are means±SD or percentages (medians in parentheses).

[2]Sum of serum eicosapentaenoic acid, docosapentaenoic acid and docosahexaenoic acid concentrations, indicated as proportion of all serum fatty acids.

SUPPLEMENTAL TABLE 5

Baseline characteristics according to fish intake among 2497 men from the Kuopio Ischaemic Heart Disease Risk Factor Study[1]

	Fish intake quartile g/d (median)			
	1<br><3 (0)	2<br>3–31 (18)	3<br>32–66 (48)	4<br>>66 (101)
Number of subjects	623	625	624	625
Age (years)	52.6±5.2 (54.3)	52.9±5.2 (54.3)	53.1±5.1 (54.3)	53.6±5.0 (54.3)*
Education (years)	8.4±3.2 (8.0)	8.9±3.5 (8.0)	8.7±3.6 (8.0)	8.5±3.4 (8.0)
Marital status, married (%)	86	88	87	88
Annual income (euro)	12985±8769 (11695)	13965±8709 (12203)	13309±8439 (11864)	13360±9955 (11525)
Body mass index (kg/m^2)	26.4±3.5 (26.0)	26.8±3.4 (26.5)	26.8±3.5 (26.4)	27.5±3.7 (27.0)*
Leisure-time physical activity (kcal/d)	151±183 (90)	136±158 (79)	129±177 (80)	149±180 (85)
Current smoker (%)	27	30	28	34*
Hypertension (%)	61	60	57	63
Coronary heart disease (%)	24	24	22	28*
Stroke (%)	2	3	3	2
Diabetes (%)	7	5	7	6
Lipid lowering medication at baseline (%)	0.6	0.2	0.8	1.0
Lipid lowering medication during follow-up (%)	49	48	51	44
Systolic blood pressure, mmHg	134±16 (131)	134±17 (131)	135±17 (133)	135±18 (133)
Diastolic blood pressure, mmHg	89±10 (89)	89±11 (88)	88±11 (88)	89±11 (88)
Serum total cholesterol, mmol/L	5.82±1.08 (5.73)	5.89±1.04 (5.86)	5.92±1.09 (5.82)	6.01±1.08 (5.93)*
Serum LDL cholesterol, mmol/L	3.91±0.98 (3.86)	4.01±1.00 (3.91)	4.09±1.06 (4.01)	4.18±1.01 (4.07)*
Serum HDL cholesterol, mmol/L	1.28±0.28 (1.25)	1.30±0.30 (1.26)	1.29±0.30 (1.25)	1.30±0.33 (1.27)
Serum triglycerides, mmol/L	1.36±0.96 (1.12)	1.35±0.79 (1.16)	1.30±0.82 (1.11)	1.25±0.72 (1.07)*
Serum long-chain omega-3 polyunsaturated fatty acids (%)[2]	3.8±1.0 (3.6)	4.4±1.1 (4.2)	4.7±1.4 (4.5)	5.7±2.0 (5.3)*
Blood glucose (mmol/L)	4.8±1.3 (4.5)	4.8±1.4 (4.5)	4.8±1.0 (4.6)	4.8±1.1 (4.6)
Serum CRP (mg/L)	2.33±4.59 (1.20)	2.45±3.81 (1.26)	2.42±4.53 (1.30)	2.50±3.60 (1.35)
Alcohol intake (g/wk)	60±136 (20)	75±162 (37)	69±113 (28)	91±118 (48)*
Dietary intakes				

Energy (kcal/d)	2443±640 (2380)	2380±580 (2345)	2396±588 (2349)	2542±665 (2504)*
Protein (E%)	14.9±2.2 (14.7)	15.4±2.3 (15.3)	15.8±2.6 (15.5)	17.0±2.6 (16.8)*
Fat (E%)	39.3±6.2 (39.1)	38.6±5.8 (38.7)	38.3±5.9 (38.4)	38.5±5.8 (38.4)*
Saturated fatty acids (E%)	18.7±4.2 (18.5)	18.2±3.9 (18.0)	18.0±4.2 (17.8)	17.8±4.0 (17.7)*
Polyunsaturated fatty acids (E%)	4.4±1.4 (4.2)	4.5±1.3 (4.3)	4.5±1.4 (4.3)	4.7±1.5 (4.5)*
Monounsaturated fatty acids (E%)	11.9±2.3 (11.8)	11.7±2.1 (11.7)	11.6±2.2 (11.4)	11.6±2.3 (11.4)*
Trans fatty acids (E%)	1.1±0.4 (1.0)	1.1±0.3 (1.0)	1.1±0.4 (1.0)	1.0±0.4 (1.0)
Cholesterol (mg/d)	390±111 (378)	396±107 (382)	397±100 (383)	422±107 (412)*
Carbohydrates (E%)	43.5±6.5 (43.9)	43.1±6.4 (42.9)	43.2±6.3 (43.2)	41.1±6.5 (41.3)*
Fiber (g/d)	25.1±7.5 (24.4)	25.0±6.8 (24.7)	25.2±7.0 (24.5)	24.9±7.2 (24.1)
Choline (mg/d)	416±89 (407)	425±84 (417)	427±81 (421)	457±92 (449)*
Phosphatidylcholine (mg/d)	185±66 (178)	191±61 (181)	183±61 (176)	192±63 (183)
Total dairy (g/d)	734±373 (705)	696±352 (677)	709±343 (682)	705±371 (674)
Fermented dairy (g/d)	176±200 (103)	174±209 (93)	202±221 (128)	203±243 (118)*
Non-fermented dairy (g/d)	559±352 (505)	521±315 (498)	507±324 (455)	502±332 (437)*
Total milk (g/d)	534±349 (485)	498±311 (468)	486±320 (433)	482±327 (418)*
Cheese (g/d)	21±26 (13)	22±24 (15)	21±24 (15)	20±26 (11)
Total meat (g/d)	174±80 (164)	165±79 (154)	147±75 (137)	151±83 (140)*
Unprocessed red meat (g/d)	76±48 (69)	73±51 (65)	65±46 (56)	62±46 (57)*
Processed red meat (g/d)	80±65 (66)	71±60 (58)	62±54 (50)	66±60 (53)*
White meat (g/d)	8±25 (0)	10±28 (0)	11±30 (0)	11±30 (0)
Game (g/d)	5±20 (0)	6±22 (0)	4±15 (0)	7±23 (0)
Offal (g/d)	5±12 (0)	5±13 (0)	5±12 (0)	5±13 (0)
Eggs (g/d)	32±27 (26)	33±24 (28)	30±23 (24)	31±24 (25)
Fish (g/d)	0±0 (0)	18±8 (18)	48±10 (48)	119±58 (101)*
Grains (g/d)	260±95 (243)	249±86 (240)	252±95 (241)	254±92 (245)
Whole grains (g/d)	160±77 (147)	155±71 (145)	157±75 (146)	164±75 (156)
Fruits, berries, and vegetables (g/d)	250±159 (228)	246±146 (223)	250±151 (227)	254±162 (225)
Potatoes (g/d)	151±90 (138)	156±85 (144)	163±86 (153)	177±90 (165)*
Fat spreads and oils (g/d)	56±24 (55)	52±22 (50)	56±25 (54)	58±26 (55)*
Butter and butter containing spreads (g/d)	36±27 (34)	33±25 (30)	36±29 (32)	37±29 (34)
Vegetable margarines (g/d)	17±18 (11)	17±15 (14)	18±17 (12)	18±18 (12)

Vegetable oils (g/d)	2±4 (1)	2±4 (1)	2±4 (1)	2±4 (0)
Tea (mL/d)	91±178 (0)	102±177 (0)	98±181 (0)	85±154 (0)
Coffee (mL/d)	568±302 (563)	559±274 (563)	559±292 (538)	569±299 (569)

**P*-trend across quartiles ≤0.05. *P*-trend was assessed with linear regression (continuous variables) or χ^2 test (bivariate relationships).

[1]Values are means±SD or percentages (medians in parentheses).

[2]Sum of serum eicosapentaenoic acid, docosapentaenoic acid and docosahexaenoic acid concentrations, indicated as proportion of all serum fatty acids.

SUPPLEMENTAL TABLE 6

Risk of Alzheimer's disease in quartiles of dairy, meat, and fish intakes among 2497 men from the Kuopio Ischaemic Heart Disease Risk Factor Study

	Intake quartile						
	1	2	3	4	*P*-trend	Per 50 g/d increase	*P* value
**Total dairy**							
Intake g/d (median)	<455 (292)	455–687 (580)	688–927 (802)	>927 (1119)			
N of events/participants	52/624 (8.3%)	66/624 (10.6%)	74/625 (11.8%)	74/624 (11.9%)			
Model 1[1]	1	1.17 (0.81, 1.68)[2]	1.17 (0.81, 1.69)	1.14 (0.77, 1.70)	0.55	1.01 (0.99, 1.03)	0.21
Model 2[3]	1	1.22 (0.84, 1.76)	1.24 (0.85, 1.80)	1.29 (0.84, 1.96)	0.26	1.02 (1.00, 1.04)	0.06
**Fermented dairy**							
Intake g/d (median)	<24 (3)	24–106 (56)	107–285 (184)	>285 (443)			
N of events/participants	73/623 (11.7%)	61/623 (9.8%)	65/627 (10.4%)	67/624 (10.7%)			
Model 1[1]	1	0.75 (0.53, 1.05)	0.78 (0.55, 1.08)	0.76 (0.55, 1.06)	0.30	1.00 (0.98, 1.03)	0.82
Model 2[3]	1	0.73 (0.52, 1.03)	0.78 (0.56, 1.10)	0.79 (0.56, 1.11)	0.47	1.01 (0.98, 1.03)	0.64
**Non-fermented dairy**							
Intake g/d (median)	<265 (158)	265–471 (372)	472–728 (585)	>728 (904)			
N of events/participants	54/624 (8.7%)	65/624 (10.4%)	78/625 (12.5%)	69/624 (11.1%)			
Model 1[1]	1	1.14 (0.79, 1.64)	1.28 (0.90, 1.83)	1.15 (0.77, 1.70)	0.45	1.01 (0.99, 1.03)	0.26
Model 2[3]	1	1.18 (0.82, 1.70)	1.37 (0.96, 1.97)	1.22 (0.81, 1.84)	0.29	1.02 (1.00, 1.04)	0.12
**Total milk**							
Intake g/d (median)	<244 (144)	244–449 (351)	450–705 (564)	>705 (875)			
N of events/participants	55/624 (8.8%)	62/624 (9.9%)	79/625 (12.6%)	70/624 (11.2%)			
Model 1[1]	1	1.05 (0.73, 1.52)	1.30 (0.91, 1.84)	1.20 (0.81, 1.77)	0.25	1.01 (0.99, 1.03)	0.23
Model 2[3]	1	1.09 (0.76, 1.58)	1.38 (0.97, 1.97)	1.28 (0.85, 1.91)	0.15	1.02 (1.00, 1.04)	0.11
**Cheese**							
Intake g/d (median)	<0 (0)	0.7–14 (8)	15–31 (21)	>31 (49)			
N of events/participants	82/709 (11.6%)	62/544 (11.4%)	65/620 (10.5%)	57/624 (9.1%)			
Model 1[1]	1	0.83 (0.59, 1.15)	0.71 (0.51, 0.98)	0.77 (0.54, 1.08)	0.15	0.81 (0.61, 1.07)	0.14
Model 2[3]	1	0.81 (0.58, 1.13)	0.73 (0.52, 1.01)	0.74 (0.51, 1.06)	0.14	0.80 (0.60, 1.08)	0.14
**Total meat**							
Intake g/d (median)	<106 (77)	106–151 (128)	152–204 (174)	>204 (261)			
N of events/participants	70/624 (11.2%)	69/625 (11.0%)	67/623 (10.8%)	60/625 (9.6%)			

Model 1[1]	1	1.01 (0.72, 1.41)	1.04 (0.74, 1.46)	1.06 (0.73, 1.53)	0.75	1.03 (0.95, 1.12)	0.52
Model 2[3]	1	0.97 (0.69, 1.36)	0.97 (0.68, 1.37)	0.94 (0.63, 1.41)	0.78	1.00 (0.91, 1.10)	0.99
**Red meat**							
Intake g/d (median)	< 91(65)	91–134 (113)	135–187 (156)	>187 (230)			
N of events/participants	72/624 (11.5%)	67/625 (10.7%)	68/624 (10.9%)	59/624 (9.5%)			
Model 1[1]	1	0.93 (0.66, 1.29)	1.00 (0.72, 1.41)	0.96 (0.66, 1.39)	0.92	1.03 (0.94, 1.12)	0.57
Model 2[3]	1	0.90 (0.64, 1.25)	0.94 (0.67, 1.33)	0.87 (0.59, 1.28)	0.55	1.00 (0.91, 1.10)	0.99
**Processed red meat**							
Intake g/d (median)	<25 (10)	25–57 (40)	58–97 (76)	>97 (139)			
N of events/participants	70/637 (11.0%)	67/608 (11.0%)	68/626 (10.9%)	61/626 (9.7%)			
Model 1[1]	1	0.94 (0.67, 1.31)	1.09 (0.77, 1.52)	1.09 (0.76, 1.56)	0.49	1.04 (0.94, 1.15)	0.48
Model 2[3]	1	0.92 (0.65, 1.29)	1.06 (0.75, 1.49)	1.01 (0.69, 1.47)	0.81	1.01 (0.90, 1.13)	0.87
**Unprocessed red meat**							
Intake g/d (median)	<39 (21)	39–67 (53)	68–103 (81)	>103 (132)			
N of events/participants	70/624 (11.2%)	70/624 (11.2%)	58/625 (9.3%)	68/624 (10.9%)			
Model 1[1]	1	0.91 (0.65, 1.27)	0.80 (0.57, 1.14)	0.93 (0.65, 1.31)	0.64	1.00 (0.88, 1.14)	0.97
Model 2[3]	1	0.93 (0.67, 1.30)	0.80 (0.56, 1.13)	0.90 (0.64, 1.28)	0.50	0.99 (0.87, 1.12)	0.84
**Fish**							
Intake g/d (median)	<3 (0)	3–31 (18)	32–66 (48)	>66 (102)			
N of events/participants	67/623 (10.8%)	73/625 (11.7%)	54/624 (8.7%)	72/625 (11.5%)			
Model 1[1]	1	0.99 (0.71, 1.38)	0.70 (0.49, 1.01)	1.01 (0.72, 1.41)	0.90	0.95 (0.84, 1.07)	0.38
Model 2[3]	1	1.01 (0.72, 1.42)	0.72 (0.50, 1.04)	0.97 (0.69, 1.37)	0.68	0.94 (0.83, 1.06)	0.30

[1]Model 1 adjusted for age, baseline examination year, and energy intake.

[2]Values are hazard ratios (95% confidence intervals).

[3]Model 2 adjusted for the Model 1 and education years, pack-years of smoking (cigarette packs/day × years of smoking), body mass index (kg/m^2), diabetes (yes/no), leisure-time physical activity (kcal/day), history of coronary heart disease (yes/no), use of lipid-lowering medication (yes/no), intakes of alcohol (g/week), fiber (g/day), sum of fruits, berries and vegetables (g/day), and dietary fat quality (ratio of polyunsaturated fatty acids plus monounsaturated fatty acids to saturated fatty acids plus trans fatty acids).

SUPPLEMENTAL TABLE 7

Frequencies of the apolipoprotein E phenotypes among 1259 men from the Kuopio Ischaemic Heart Disease Risk Factor Study

Phenotype	Frequency (n)	Proportion (%)
2/2	4	0.3
2/3	75	6.0
3/3	745	59.2
2/4	17	1.4
3/4	373	29.6
4/4	45	3.6

SUPPLEMENTAL TABLE 8

Complete case analysis of the risk of dementia in quartiles of dairy, meat, and fish intakes among 2416 men from the Kuopio Ischaemic Heart Disease Risk Factor Study

	Intake quartile						
	1	2	3	4	*P*-trend	Per 50 g/d increase	*P* value
**Total dairy**							
Intake, g/d (median)	<459 (287)	459–690 (579)	691–928 (805)	>928 (1187)			
N of events/participants	70/604 (11.6%)	83/604 (13.7%)	87/604 (14.4%)	90/604 (14.9%)			
Model 1[a]	1	1.06 (0.77, 1.47)[b]	1.07 (0.77, 1.48)	1.13 (0.79, 1.60)	0.52	1.01 (0.99, 1.03)	0.16
Model 2[c]	1	1.13 (0.82, 1.56)	1.11 (0.80, 1.55)	1.27 (0.87, 1.84)	0.25	1.02 (1.00, 1.04)	0.05
**Fermented dairy**							
Intake, g/d (median)	<24 (7)	24–107 (61)	108–285 (188)	>285 (506)			
N of events/participants	91/603 (15.1%)	75/605 (12.4%)	82/604 (13.6%)	82/604 (13.6%)			
Model 1[a]	1	0.75 (0.55, 1.02)	0.79 (0.59, 1.07)	0.78 (0.58, 1.05)	0.31	1.00 (0.98, 1.02)	0.98
Model 2[c]	1	0.75 (0.55, 1.03)	0.83 (0.61, 1.13)	0.84 (0.62, 1.14)	0.64	1.00 (0.98, 1.03)	0.71
**Non-fermented dairy**							
Intake, g/d (median)	<267 (152)	267–473 (373)	474–730 (593)	>730 (978)			
N of events/participants	73/604 (12.1%)	75/604 (12.4%)	100/604 (16.6%)	82/604 (13.6%)			
Model 1[a]	1	0.99 (0.72, 1.37)	1.29 (0.94, 1.75)	1.11 (0.78, 1.57)	0.34	1.01 (0.99, 1.03)	0.15
Model 2[c]	1	1.03 (0.74, 1.43)	1.38 (1.01, 1.89)	1.13 (0.79, 1.63)	0.29	1.02 (1.00, 1.04)	0.09
**Total milk**							
Intake, g/d (median)	<246 (136)	246–450 (350)	451–710 (570)	>710 (952)			
N of events/participants	75/604 (12.4%)	74/604 (12.3%)	97/604 (16.1%)	84/604 (13.9%)			
Model 1[a]	1	0.95 (0.69, 1.31)	1.22 (0.89, 1.65)	1.15 (0.81, 1.61)	0.24	1.01 (0.99, 1.03)	0.16
Model 2[c]	1	0.98 (0.71, 1.36)	1.29 (0.94, 1.77)	1.16 (0.81, 1.66)	0.21	1.02 (1.00, 1.04)	0.11
**Cheese**							
Intake, g/d (median)	<0 (0)	0–13 (8)	14–31 (21)	>31 (55)			
N of events/participants	105/689 (15.2%)	77/519 (14.8%)	77/604 (12.7%)	71/604 (11.8%)			
Model 1[a]	1	0.83 (0.62, 1.11)	0.66 (0.49, 0.88)	0.74 (0.55, 1.01)	0.06	0.80 (0.62, 1.03)	0.09
Model 2[c]	1	0.82 (0.61, 1.11)	0.69 (0.51, 0.94)	0.72 (0.52, 1.00)	0.06	0.80 (0.62, 1.05)	0.11
**Total meat**							
Intake, g/d (median)	<104 (70)	104–148 (126)	149–203 (174)	>203 (268)			
N of events/participants	86/604 (14.2%)	86/604 (14.2%)	88/604 (14.6%)	70/604 (11.6%)			

Model 1[a]	1	1.08 (0.79, 1.45)	1.16 (0.85, 1.57)	1.09 (0.78, 1.54)	0.54	1.04 (0.97, 1.12)	0.29
Model 2[c]	1	1.05 (0.77, 1.42)	1.10 (0.80, 1.50)	0.99 (0.69, 1.42)	0.99	1.02 (0.93, 1.11)	0.73
**Red meat**							
Intake, g/d (median)	< 91(59)	91–134 (112)	135–188 (158)	>188 (248)			
N of events/participants	88/604 (14.6%)	88/604 (14.6%)	82/604 (13.6%)	72/604 (11.9%)			
Model 1[a]	1	1.04 (0.77, 1.40)	1.04 (0.76, 1.41)	1.05 (0.75, 1.47)	0.80	1.03 (0.95, 1.12)	0.41
Model 2[c]	1	1.02 (0.76, 1.38)	0.97 (0.71, 1.33)	0.95 (0.67, 1.35)	0.73	1.00 (0.92, 1.10)	0.92
**Processed red meat**							
Intake, g/d (median)	<25 (10)	25–57 (41)	58–97 (76)	>97 (153)			
N of events/participants	83/619 (13.4%)	88/585 (15.0%)	82/606 (13.5%)	77/606 (12.7%)			
Model 1[a]	1	1.09 (0.80, 1.47)	1.17 (0.86, 1.59)	1.24 (0.90, 1.72)	0.17	1.07 (0.98, 1.18)	0.13
Model 2[c]	1	1.06 (0.79, 1.44)	1.15 (0.84, 1.57)	1.14 (0.81, 1.60)	0.43	1.04 (0.94, 1.15)	0.42
**Unprocessed red meat**							
Intake, g/d (median)	<39 (20)	39–67 (53)	68–103 (82)	>103 (143)			
N of events/participants	89/604 (14.7%)	87/604 (14.4%)	76/604 (12.6%)	78/604 (12.9%)			
Model 1[a]	1	0.91 (0.68, 1.23)	0.85 (0.63, 1.16)	0.87 (0.63, 1.19)	0.37	0.96 (0.86, 1.08)	0.54
Model 2[c]	1	0.93 (0.69, 1.26)	0.84 (0.62, 1.15)	0.85 (0.62, 1.16)	0.27	0.95 (0.85, 1.07)	0.44
**Fish**							
Intake, g/d (median)	<3 (0)	3–31 (18)	32–65 (48)	>65 (118)			
N of events/participants	75/604 (12.4%)	92/604 (15.2%)	70/604 (11.6%)	93/604 (15.4%)			
Model 1[a]	1	1.13 (0.83, 1.53)	0.83 (0.60, 1.15)	1.21 (0.89, 1.64)	0.37	1.01 (0.91, 1.12)	0.86
Model 2[c]	1	1.15 (0.84, 1.57)	0.84 (0.61, 1.17)	1.14 (0.84, 1.56)	0.68	0.99 (0.89, 1.10)	0.89

[a]Model 1 adjusted for age, baseline examination year, and energy intake.

[b]Values are hazard ratios (95% confidence intervals).

[c]Model 2 adjusted for the Model 1 and education years, pack-years of smoking (cigarette packs/day × years of smoking), body mass index (kg/m^2), diabetes (yes/no), leisure-time physical activity (kcal/day), history of coronary heart disease (yes/no), use of lipid-lowering medication (yes/no), intakes of alcohol (g/week), fiber (g/day), sum of fruits, berries and vegetables (g/day), and dietary fat quality (ratio of polyunsaturated fatty acids plus monounsaturated fatty acids to saturated fatty acids plus trans fatty acids).

SUPPLEMENTAL TABLE 9

Complete case analysis of the risk of Alzheimer's disease in quartiles of dairy, meat, and fish intakes among 2416 men from the Kuopio Ischaemic Heart Disease Risk Factor Study

	Intake quartile						
	1	2	3	4	*P*-trend	Per 50 g/d increase	*P* value
**Total dairy**							
Intake, g/d (median)	<459 (287)	459–690 (579)	691–928 (805)	>928 (1187)			
N of events/participants	54/604 (8.9%)	61/604 (10.1%)	71/604 (11.8%)	73/604 (12.1%)			
Model 1[a]	1	1.00 (0.69, 1.45)[b]	1.10 (0.76, 1.59)	1.12 (0.75, 1.67)	0.50	1.02 (0.99, 1.04)	0.16
Model 2[c]	1	1.04 (0.72, 1.52)	1.17 (0.81, 1.70)	1.27 (0.83, 1.93)	0.22	1.02 (1.00, 1.05)	0.04
**Fermented dairy**							
Intake, g/d (median)	<24 (7)	24–107 (61)	108–285 (188)	>285 (506)			
N of events/participants	71/603 (11.8%)	60/605 (9.9%)	63/604 (10.4%)	65/604 (10.8%)			
Model 1[a]	1	0.74 (0.53, 1.05)	0.77 (0.55, 1.08)	0.77 (0.55, 1.08)	0.35	1.00 (0.98, 1.03)	0.79
Model 2[c]	1	0.72 (0.51, 1.02)	0.78 (0.55, 1.09)	0.81 (0.57, 1.14)	0.58	1.01 (0.98, 1.04)	0.57
**Non-fermented dairy**							
Intake, g/d (median)	<267 (152)	267–473 (372)	474–730 (593)	>730 (978)			
N of events/participants	55/604 (9.1%)	61/604 (10.1%)	76/604 (12.6%)	67/604 (11.1%)			
Model 1[a]	1	1.05 (0.73, 1.52)	1.26 (0.88, 1.80)	1.14 (0.77, 1.69)	0.40	1.01 (0.99, 1.03)	0.22
Model 2[c]	1	1.08 (0.75, 1.57)	1.34 (0.94, 1.93)	1.21 (0.80, 1.82)	0.26	1.02 (1.00, 1.04)	0.10
**Total milk**							
Intake, g/d (median)	<246 (136)	246–450 (350)	451–710 (570)	>710 (952)			
N of events/participants	55/604 (9.1%)	60/604 (9.9%)	75/604 (12.4%)	69/604 (11.4%)			
Model 1[a]	1	1.04 (0.72, 1.50)	1.25 (0.88, 1.78)	1.23 (0.83, 1.82)	0.20	1.01 (0.99, 1.04)	0.20
Model 2[c]	1	1.07 (0.74, 1.54)	1.32 (0.92, 1.90)	1.30 (0.87, 1.95)	0.13	1.02 (1.00, 1.04)	0.10
**Cheese**							
Intake, g/d (median)	<0 (0)	0–13 (8)	14–31 (21)	>31 (55)			
N of events/participants	80/689 (11.6%)	59/519 (11.4%)	63/604 (10.4%)	57/604 (9.4%)			
Model 1[a]	1	0.83 (0.59, 1.16)	0.69 (0.49, 0.96)	0.76 (0.54, 1.07)	0.14	0.81 (0.61, 1.08)	0.15
Model 2[c]	1	0.81 (0.57, 1.14)	0.71 (0.50, 0.99)	0.73 (0.51, 1.06)	0.13	0.81 (0.60, 1.09)	0.16
**Total meat**							
Intake, g/d (median)	<104 (70)	104–148 (126)	149–203 (174)	>203 (268)			
N of events/participants	67/604 (11.1%)	67/604 (11.1%)	68/604 (11.3%)	57/604 (9.4%)			

Model 1[a]	1	1.05 (0.75, 1.48)	1.11 (0.79, 1.57)	1.08 (0.74, 1.58)	0.65	1.04 (0.96, 1.13)	0.37
Model 2[c]	1	1.00 (0.71, 1.41)	1.03 (0.72, 1.46)	0.94 (0.63, 1.42)	0.80	1.01 (0.91, 1.11)	0.90
**Red meat**							
Intake, g/d (median)	< 91(59)	91–134 (112)	135–188 (158)	>188 (248)			
N of events/participants	69/604 (11.4%)	67/604 (11.1%)	65/604 (10.8%)	58/604 (9.6%)			
Model 1[a]	1	0.98 (0.70, 1.38)	1.02 (0.72, 1.43)	1.01 (0.69, 1.47)	0.93	1.04 (0.95, 1.13)	0.43
Model 2[c]	1	0.94 (0.67, 1.32)	0.95 (0.67, 1.35)	0.90 (0.61, 1.34)	0.63	1.01 (0.91, 1.11)	0.90
**Processed red meat**							
Intake, g/d (median)	<25 (10)	25–57 (41)	58–97 (76)	>97 (153)			
N of events/participants	68/619 (11.0%)	64/585 (10.9%)	67/606 (11.1%)	60/606 (9.9%)			
Model 1[a]	1	0.94 (0.67, 1.33)	1.13 (0.80, 1.59)	1.13 (0.79, 1.63)	0.35	1.05 (0.95, 1.17)	0.35
Model 2[c]	1	0.91 (0.65, 1.29)	1.10 (0.77, 1.55)	1.03 (0.70, 1.51)	0.69	1.02 (0.91, 1.14)	0.75
**Unprocessed red meat**							
Intake, g/d (median)	<39 (20)	39–67 (53)	68–103 (82)	>103 (143)			
N of events/participants	69/604 (11.4%)	66/604 (10.9%)	55/604 (9.1%)	69/604 (11.4%)			
Model 1[a]	1	0.88 (0.63, 1.24)	0.78 (0.55, 1.12)	0.96 (0.68, 1.36)	0.84	1.00 (0.88, 1.14)	0.99
Model 2[c]	1	0.90 (0.64, 1.27)	0.77 (0.54, 1.10)	0.93 (0.66, 1.32)	0.67	0.99 (0.87, 1.13)	0.85
**Fish**							
Intake, g/d (median)	<3 (0)	3–31 (18)	32–65 (48)	>65 (118)			
N of events/participants	64/604 (10.6%)	71/604 (11.8%)	53/604 (8.8%)	71/604 (11.8%)			
Model 1[a]	1	1.01 (0.72, 1.42)	0.73 (0.51, 1.05)	1.06 (0.76, 1.49)	0.86	0.95 (0.84, 1.07)	0.41
Model 2[c]	1	1.04 (0.74, 1.47)	0.76 (0.52, 1.09)	1.03 (0.73, 1.45)	0.90	0.94 (0.83, 1.06)	0.32

[a]Model 1 adjusted for age, baseline examination year, and energy intake.

[b]Values are hazard ratios (95% confidence intervals).

[c]Model 2 adjusted for the Model 1 and education years, pack-years of smoking (cigarette packs/day × years of smoking), body mass index (kg/m^2), diabetes (yes/no), leisure-time physical activity (kcal/day), history of coronary heart disease (yes/no), use of lipid-lowering medication (yes/no), intakes of alcohol (g/week), fiber (g/day), sum of fruits, berries and vegetables (g/day), and dietary fat quality (ratio of polyunsaturated fatty acids plus monounsaturated fatty acids to saturated fatty acids plus trans fatty acids).

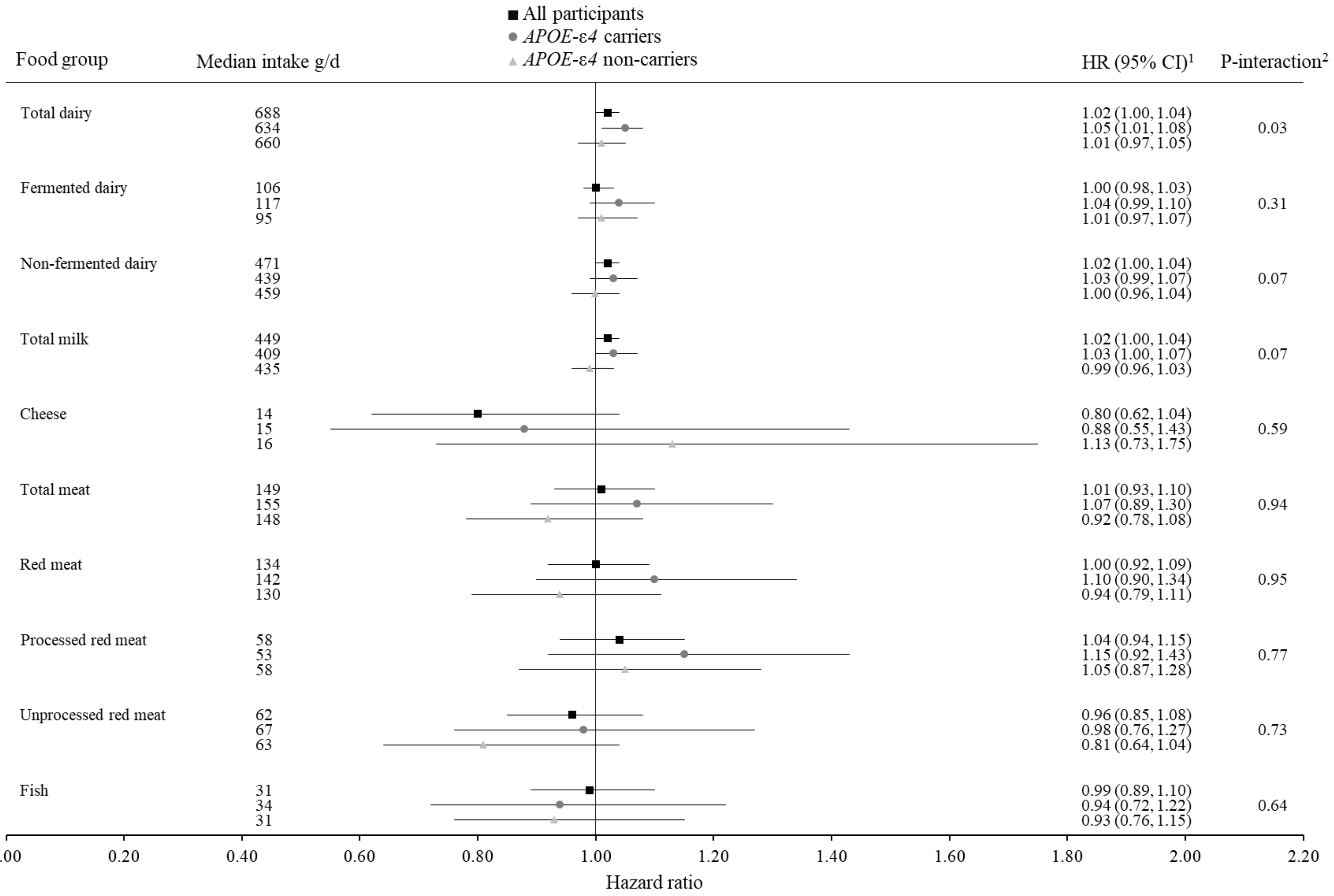

SUPPLEMENTAL FIGURE 2

Risk of dementia per 50 g/d intake increase stratified by the apolipoprotein E ε4 phenotype among 1259 men from the Kuopio Ischaemic Heart Disease Risk Factor Study.

[1]Values are hazard ratios (95% confidence intervals) adjusted for age, baseline examination year, and energy intake, education years, pack-years of smoking (cigarette packs/day × years of smoking), body mass index (kg/m^2), diabetes (yes/no), leisure-time physical activity (kcal/day), history of coronary heart disease (yes/no), use of lipid-lowering medication (yes/no), intakes of alcohol (g/week), fiber (g/day), sum of fruits, berries and vegetables (g/day), and dietary fat quality (ratio of polyunsaturated fatty acids plus monounsaturated fatty acids to saturated fatty acids plus trans fatty acids).

[2]intake × *APOE ε4* carrier status

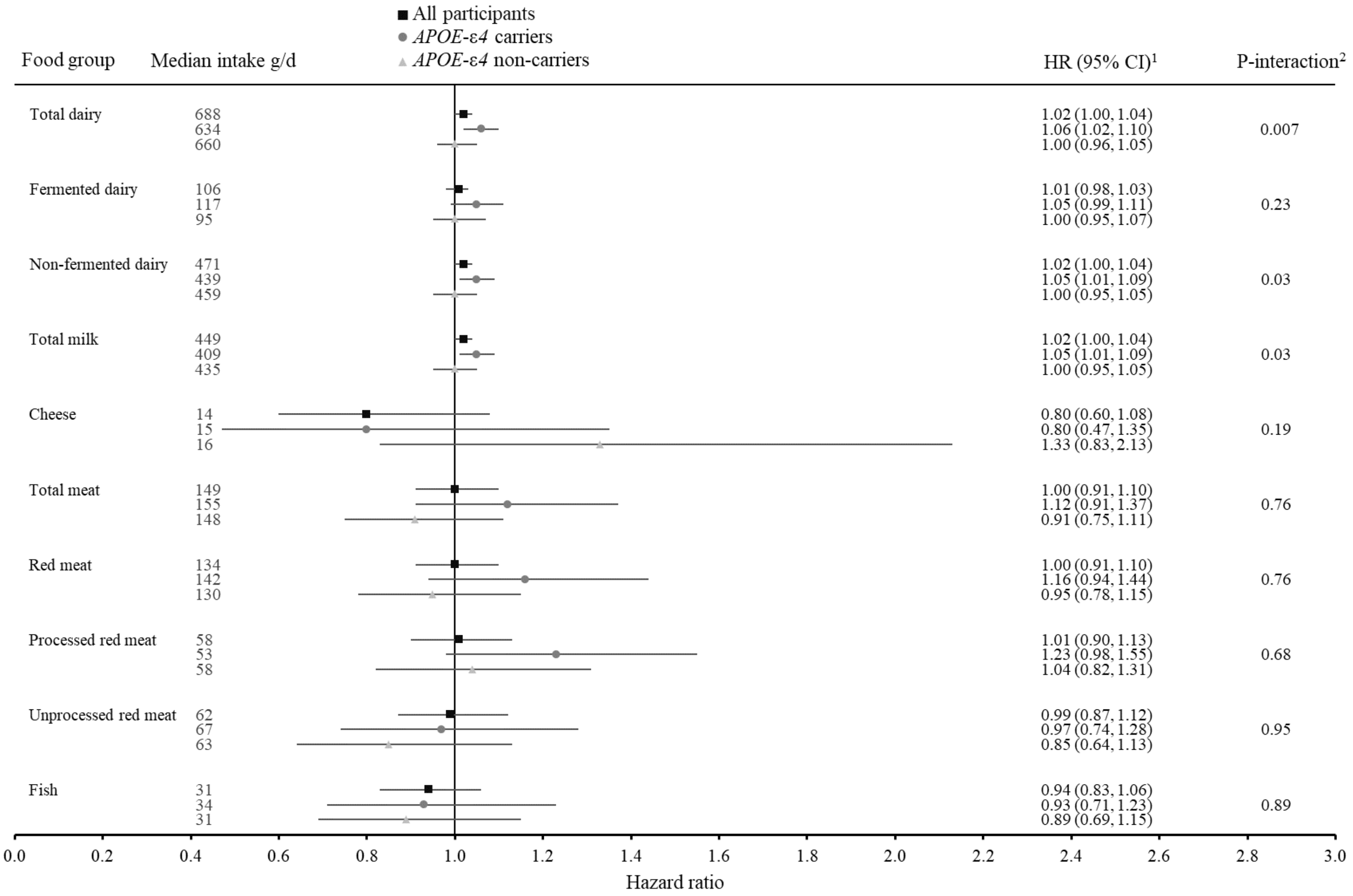

SUPPLEMENTAL FIGURE 3

Risk of Alzheimer's disease per 50 g/d intake increase stratified by the apolipoprotein E ε4 phenotype among 1259 men from the Kuopio Ischaemic Heart Disease Risk Factor Study.

[1]Values are hazard ratios (95% confidence intervals) adjusted for age, baseline examination year, and energy intake, education years, pack-years of smoking (cigarette packs/day × years of smoking), body mass index (kg/m^2), diabetes (yes/no), leisure-time physical activity (kcal/day), history of coronary heart disease (yes/no), use of lipid-lowering medication (yes/no), intakes of alcohol (g/week), fiber (g/day), sum of fruits, berries and vegetables (g/day), and dietary fat quality (ratio of polyunsaturated fatty acids plus monounsaturated fatty acids to saturated fatty acids plus trans fatty acids).

[2]intake × *APOE ε4* carrier status

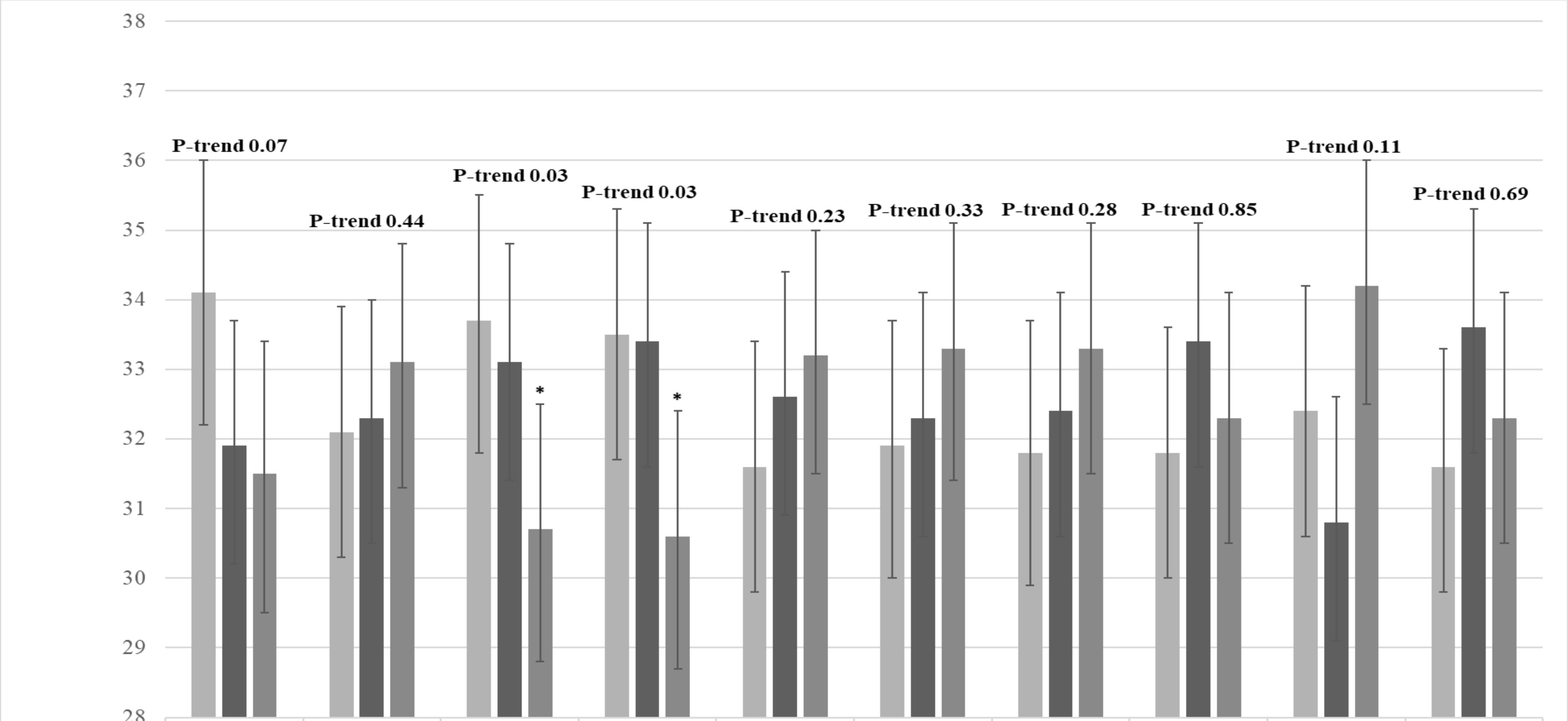

	Total dairy	Fermented dairy	Non-fermented dairy	Total milk	Cheese	Total meat	Red meat	Processed red meat	Unprocessed red meat	Fish
1st tertile	34.1	32.1	33.7	33.5	31.6	31.9	31.8	31.8	32.4	31.6
2nd tertile	31.9	32.3	33.1	33.4	32.6	32.3	32.4	33.4	30.8	33.6
3rd tertile	31.5	33.1	30.7	30.6	33.2	33.3	33.3	32.3	34.2	32.3

SUPPLEMENTAL FIGURE 4

Cognitive performance in the Verbal Fluency Test after 4 y of follow up in tertiles of baseline dairy, meat, and fish intakes among 482 men from the Kuopio Ischaemic Heart Disease Risk Factor Study.

The bars and the values under the figure show the mean number of words (the error bars illustrating the 95% CIs). Values were obtained by ANCOVA and were adjusted for age, baseline examination year, and energy intake, education years, pack-years of smoking (cigarette packs/day × years of smoking), body mass index (kg/m^2), diabetes (yes/no), leisure-time physical activity (kcal/day), history of coronary heart disease (yes/no), use of lipid-lowering medication (yes/no), intakes of alcohol (g/week), fiber (g/day), sum of fruits, berries and vegetables (g/day), and dietary fat quality (ratio of polyunsaturated fatty acids plus monounsaturated fatty acids to saturated fatty acids plus trans fatty acids).

The intakes in the tertiles (medians in the parentheses) were <538 (353) g/d, 538–804 (663) g/d, and >804 (970) g/d for total dairy; <45 (10) g/d, 45–227 (115) g/d, and >227 (375) g/d for fermented dairy; <307 (185) g/d, 307–594 (455) g/d, and >594 (797) g/d for non-fermented dairy; <279 (165) g/d, 279–571 (433) g/d, and >571 (764) g/d for total milk; <5 (0) g/d, 5–25 (13) g/d, and >25 (40) g/d for cheese; <111 (78) g/d, 111–171 (146) g/d, and >171 (210) g/d for total meat; <96 (68) g/d, 96–155 (125) g/d, and >155 (199) g/d for red meat; <31 (13) g/d, 31–65 (45) g/d, and >65 (101) g/d for processed red meat; <48 (27) g/d, 48–87 (67) g/d, and >87 (121) g/d for unprocessed red meat; and <18 (0) g/d, 18–55 (36) g/d, and >55 (89) g/d for fish, respectively.

* $P<0.05$ compared to the 1st tertile.

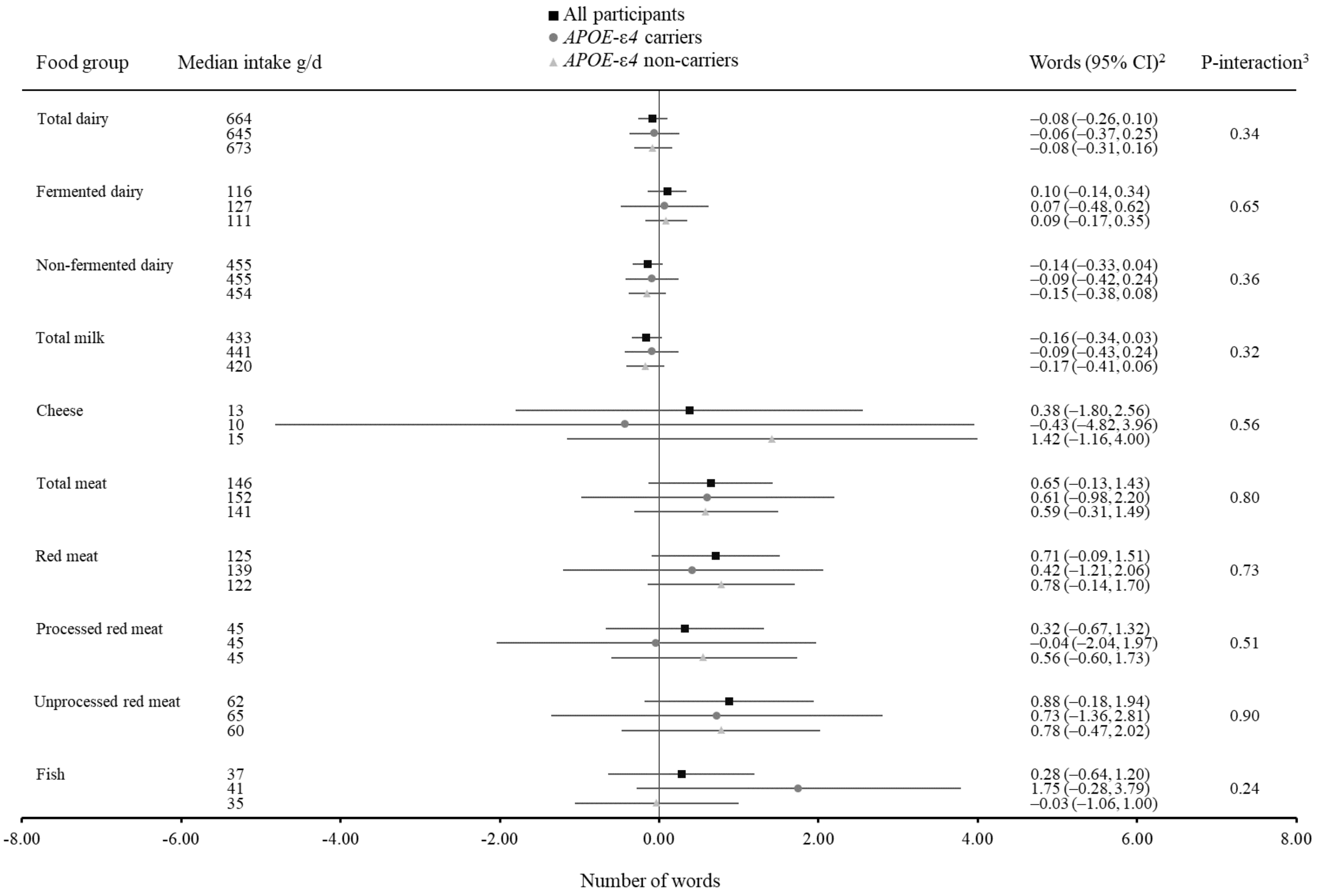

SUPPLEMENTAL FIGURE 5

Cognitive performance in the Verbal Fluency Test after 4 y of follow-up per 50 g/d increase of baseline dairy, meat, and fish intakes stratified by the apolipoprotein E ε4 phenotype among 480[1] men from the Kuopio Ischaemic Heart Disease Risk Factor Study.

[1]482 men among all participants

[2]Values were obtained by ANCOVA and were adjusted for age, baseline examination year, and energy intake, education years, pack-years of smoking (cigarette packs/day × years of smoking), body mass index (kg/m^2), diabetes (yes/no), leisure-time physical activity (kcal/day), history of coronary heart disease (yes/no), use of lipid-lowering medication (yes/no), intakes of alcohol (g/week), fiber (g/day), sum of fruits, berries and vegetables (g/day), and dietary fat quality (ratio of polyunsaturated fatty acids plus monounsaturated fatty acids to saturated fatty acids plus trans fatty acids).

[3]intake × *APOE ε4* carrier status

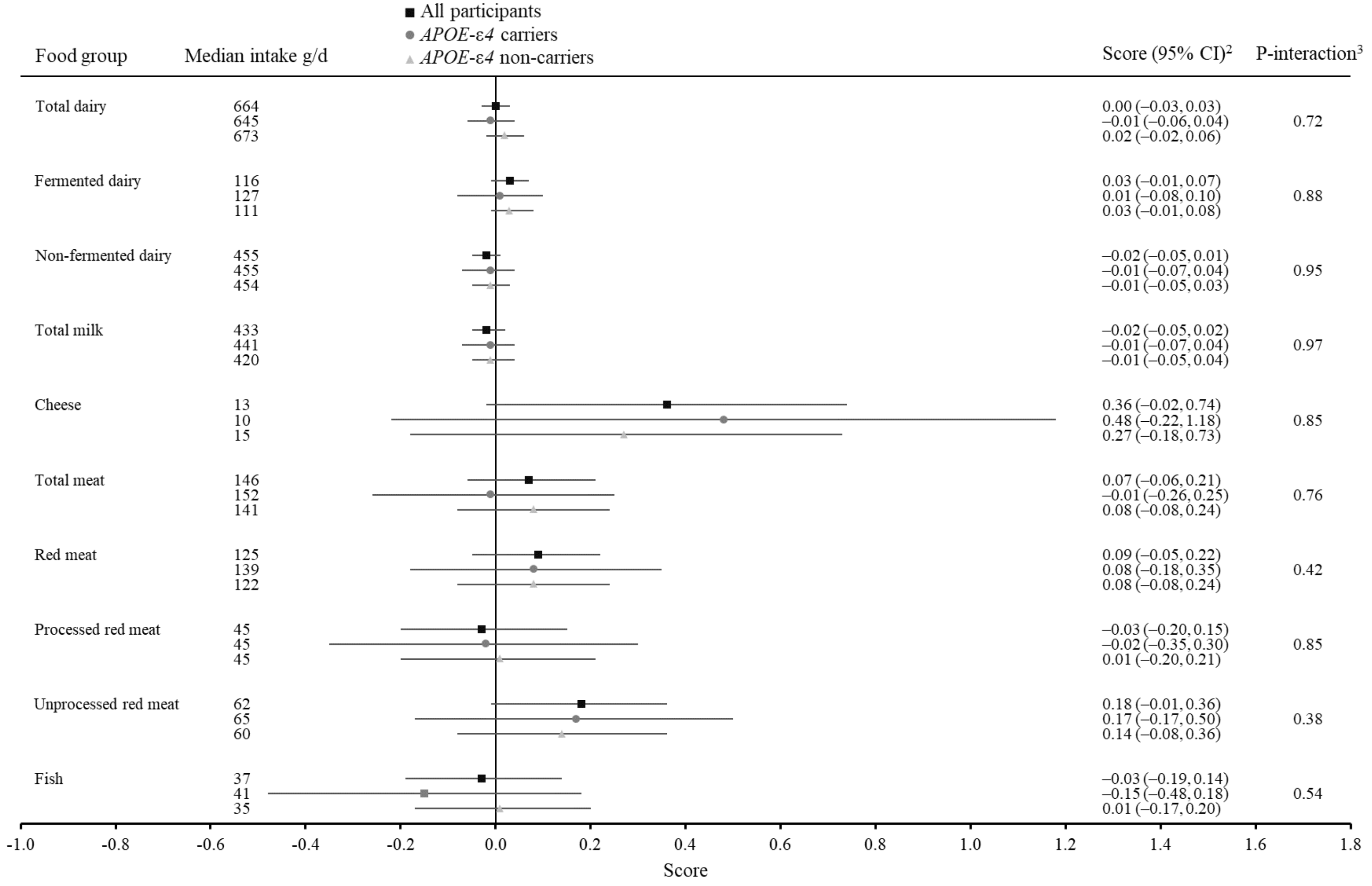

SUPPLEMENTAL FIGURE 6

Cognitive performance in the Mini Mental State Exam after 4 y of follow-up per 50 g/d increase of baseline dairy, meat, and fish intakes stratified by the apolipoprotein E ε4 phenotype among 480[1] men from the Kuopio Ischaemic Heart Disease Risk Factor Study.

[1]482 men among all participants

[2]Values were obtained by ANCOVA and were adjusted for age, baseline examination year, and energy intake, education years, pack-years of smoking (cigarette packs/day × years of smoking), body mass index (kg/m^2), diabetes (yes/no), leisure-time physical activity (kcal/day), history of coronary heart disease (yes/no), use of lipid-lowering medication (yes/no), intakes of alcohol (g/week), fiber (g/day), sum of fruits, berries and vegetables (g/day), and dietary fat quality (ratio of polyunsaturated fatty acids plus monounsaturated fatty acids to saturated fatty acids plus trans fatty acids).

[3]intake × *APOE ε4* carrier status

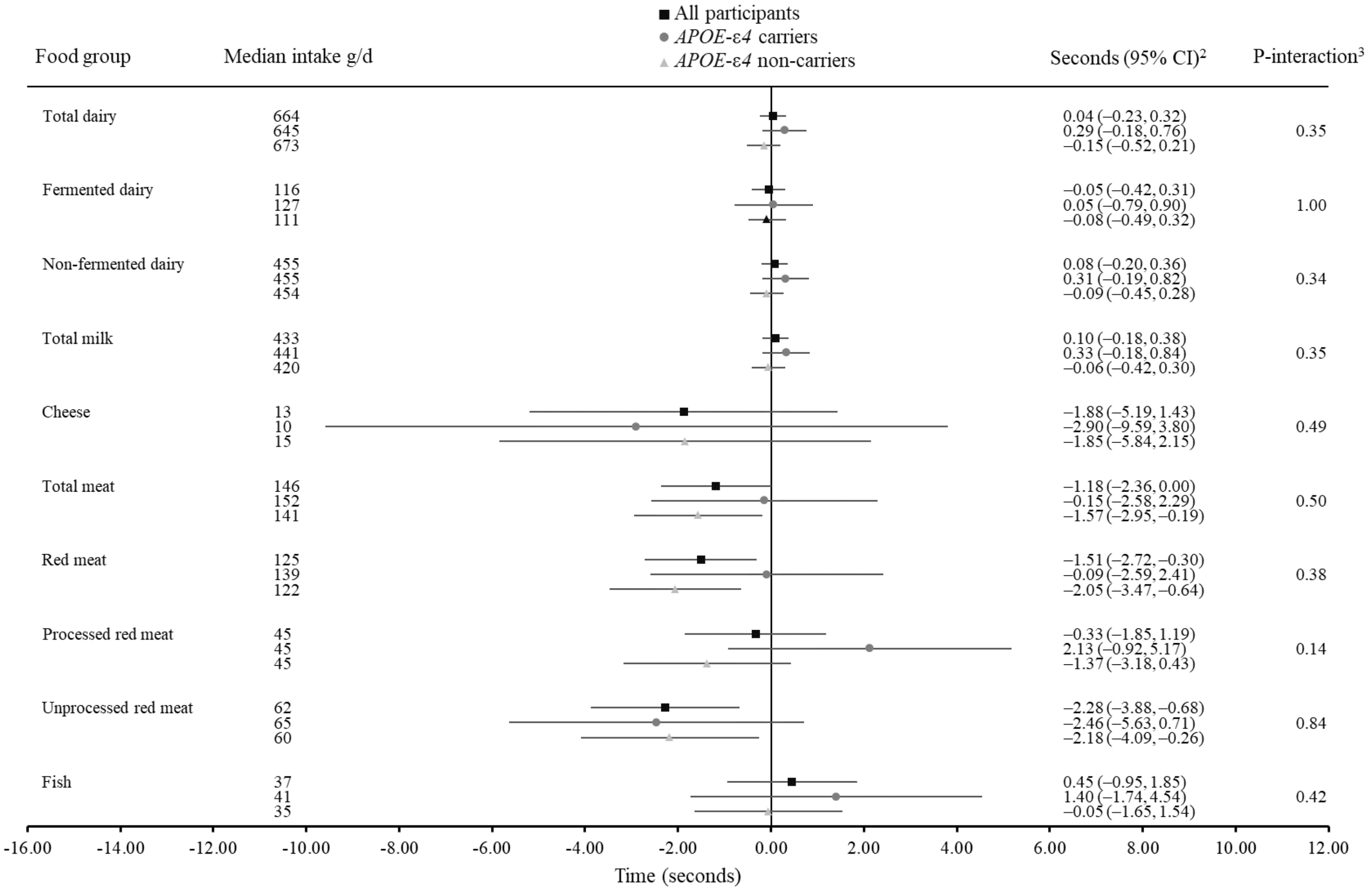

SUPPLEMENTAL FIGURE 7

Cognitive performance in the Trail Making Test after 4 y of follow-up per 50 g/d increase of baseline dairy, meat, and fish intakes stratified by the apolipoprotein E ε4 phenotype among 480[1] men from the Kuopio Ischaemic Heart Disease Risk Factor Study.

[1]482 men among all participants

[2]Values were obtained by ANCOVA and were adjusted for age, baseline examination year, and energy intake, education years, pack-years of smoking (cigarette packs/day × years of smoking), body mass index (kg/m^2), diabetes (yes/no), leisure-time physical activity (kcal/day), history of coronary heart disease (yes/no), use of lipid-lowering medication (yes/no), intakes of alcohol (g/week), fiber (g/day), sum of fruits, berries and vegetables (g/day), and dietary fat quality (ratio of polyunsaturated fatty acids plus monounsaturated fatty acids to saturated fatty acids plus trans fatty acids).

[3]intake × *APOE ε4* carrier status

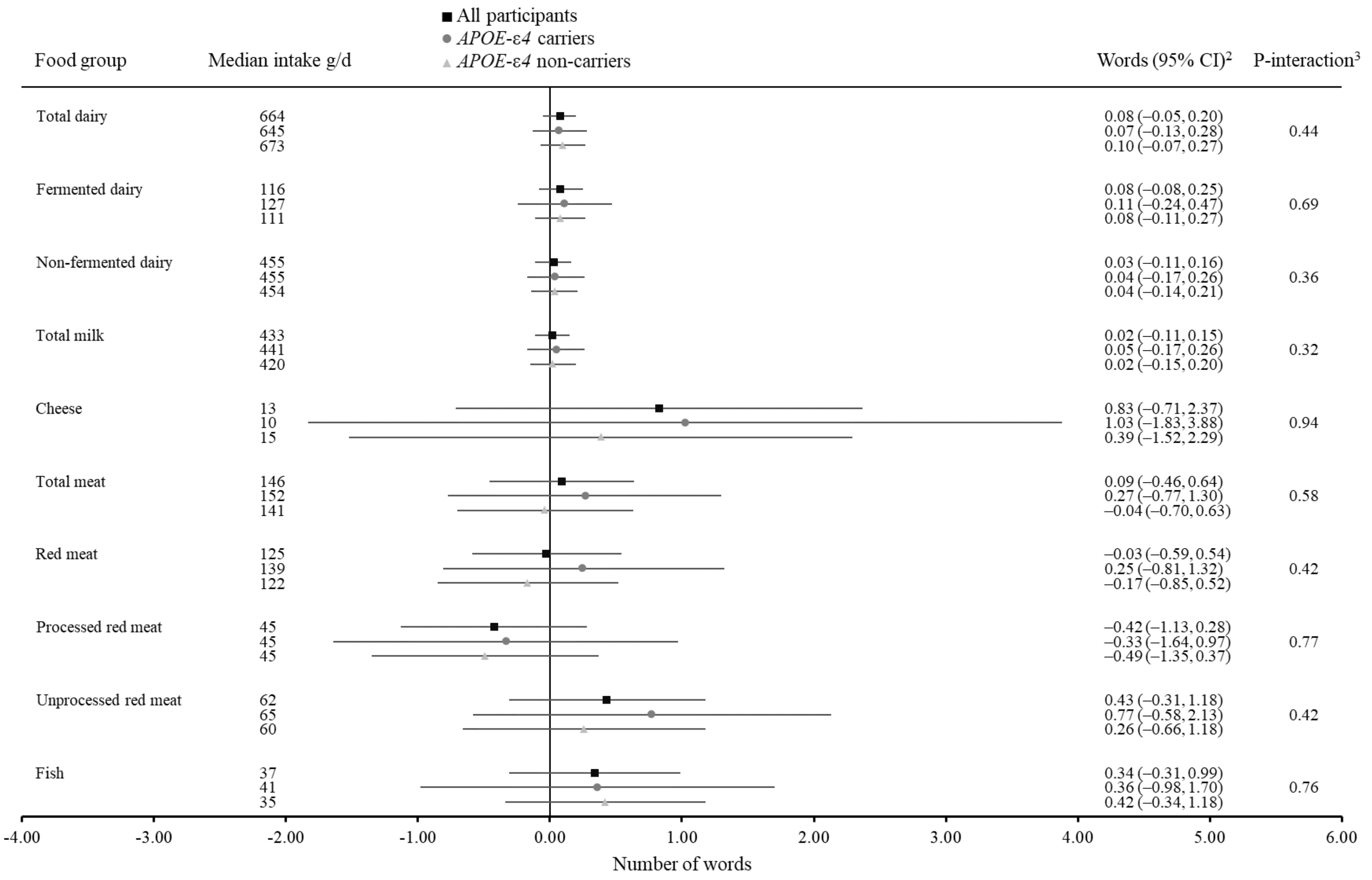

SUPPLEMENTAL FIGURE 8

Cognitive performance in the Selective Reminding Test after 4 y of follow-up per 50 g/d increase of baseline dairy, meat, and fish intakes stratified by the apolipoprotein E ε4 phenotype among 480[1] men from the Kuopio Ischaemic Heart Disease Risk Factor Study.

[1]482 men among all participants

[2]Values were obtained by ANCOVA and were adjusted for age, baseline examination year, and energy intake, education years, pack-years of smoking (cigarette packs/day × years of smoking), body mass index (kg/m^2), diabetes (yes/no), leisure-time physical activity (kcal/day), history of coronary heart disease (yes/no), use of lipid-lowering medication (yes/no), intakes of alcohol (g/week), fiber (g/day), sum of fruits, berries and vegetables (g/day), and dietary fat quality (ratio of polyunsaturated fatty acids plus monounsaturated fatty acids to saturated fatty acids plus trans fatty acids).

[3]intake × *APOE ε4* carrier status

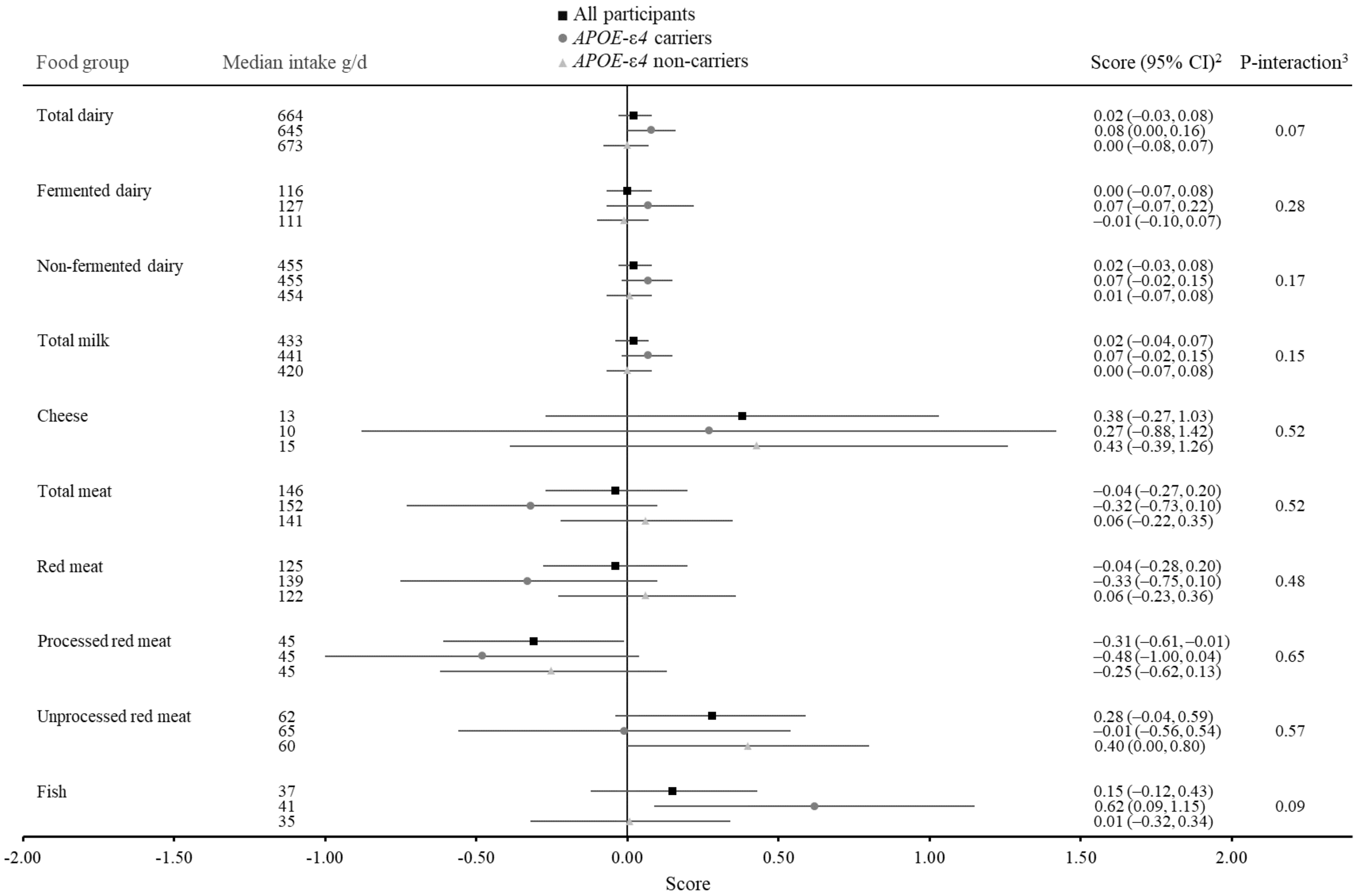

SUPPLEMENTAL FIGURE 9

Cognitive performance in the Russell's adaptation of the Visual Reproduction Test after 4 y of follow-up per 50 g/d increase of baseline dairy, meat, and fish intakes stratified by the apolipoprotein E ε4 phenotype among 480[1] men from the Kuopio Ischaemic Heart Disease Risk Factor Study.

[1]482 men among all participants

[2]Values were obtained by ANCOVA and were adjusted for age, baseline examination year, and energy intake, education years, pack-years of smoking (cigarette packs/day × years of smoking), body mass index (kg/m^2), diabetes (yes/no), leisure-time physical activity (kcal/day), history of coronary heart disease (yes/no), use of lipid-lowering medication (yes/no), intakes of alcohol (g/week), fiber (g/day), sum of fruits, berries and vegetables (g/day), and dietary fat quality (ratio of polyunsaturated fatty acids plus monounsaturated fatty acids to saturated fatty acids plus trans fatty acids).

[3]intake × *APOE ε4* carrier status

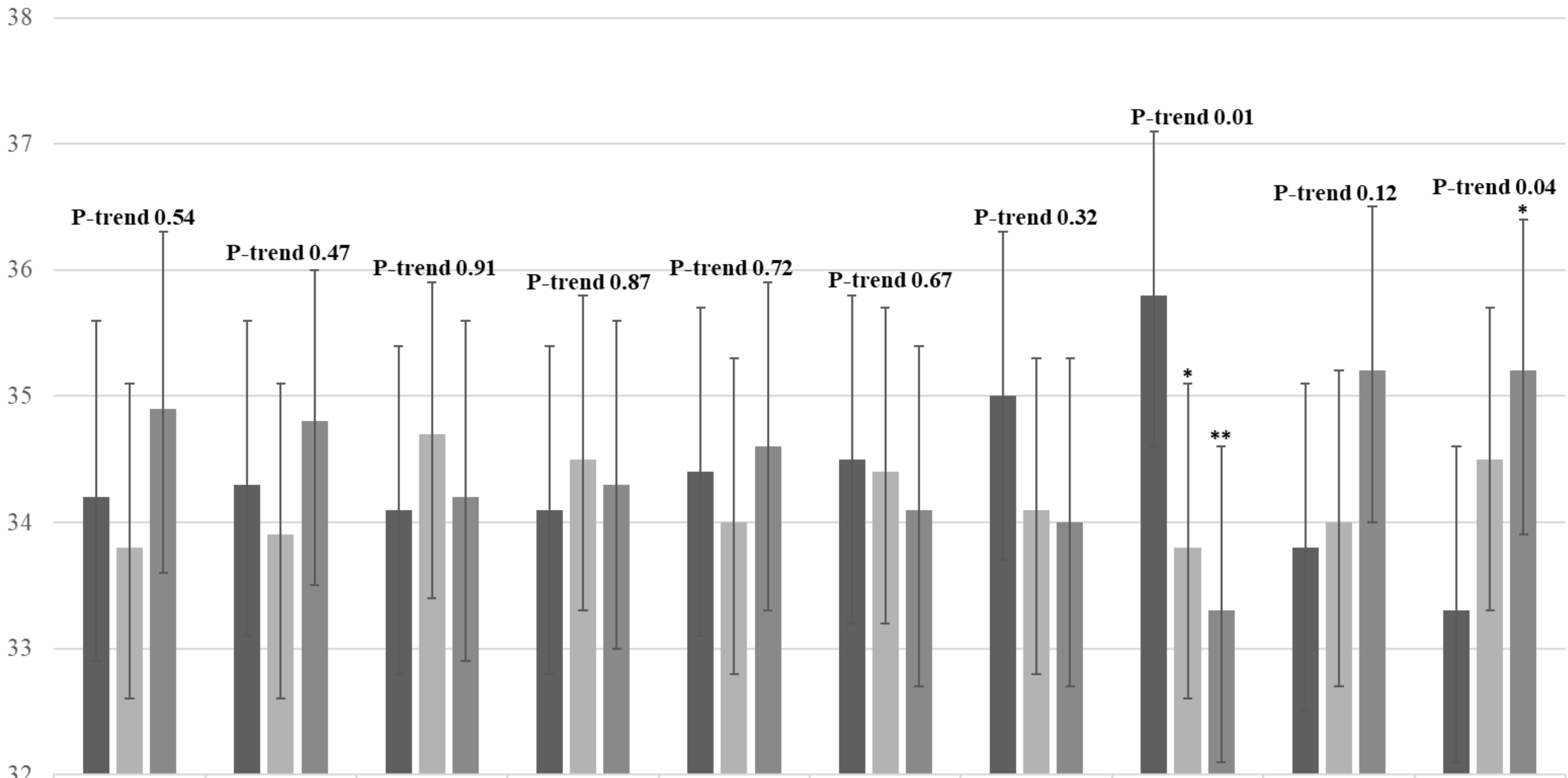

	Total dairy	Fermented dairy	Non-fermented dairy	Total milk	Cheese	Total meat	Red meat	Processed red meat	Unprocessed red meat	Fish
■ 1st tertile	34.2	34.3	34.1	34.1	34.4	34.5	35.0	35.8	33.8	33.3
■ 2nd tertile	33.8	33.9	34.7	34.5	34.0	34.4	34.1	33.8	34.0	34.5
■ 3rd tertile	34.9	34.8	34.2	34.3	34.6	34.1	34.0	33.3	35.2	35.2

SUPPLEMENTAL FIGURE 10

Cognitive performance in the Selective Reminding Test after 4 y of follow up in tertiles of baseline dairy, meat, and fish intakes among 482 men from the Kuopio Ischaemic Heart Disease Risk Factor Study.

The bars and the values under the figure show the mean number of words (the error bars illustrating the 95% CIs). Values were obtained by ANCOVA and were adjusted for age, baseline examination year, and energy intake, education years, pack-years of smoking (cigarette packs/day × years of smoking), body mass index (kg/m^2), diabetes (yes/no), leisure-time physical activity (kcal/day), history of coronary heart disease (yes/no), use of lipid-lowering medication (yes/no), intakes of alcohol (g/week), fiber (g/day), sum of fruits, berries and vegetables (g/day), and dietary fat quality (ratio of polyunsaturated fatty acids plus monounsaturated fatty acids to saturated fatty acids plus trans fatty acids).

The intakes in the tertiles (medians in the parentheses) were <538 (353) g/d, 538–804 (663) g/d, and >804 (970) g/d for total dairy; <45 (10) g/d, 45–227 (115) g/d, and >227 (375) g/d for fermented dairy; <307 (185) g/d, 307–594 (455) g/d, and >594 (797) g/d for non-fermented dairy; <279 (165) g/d, 279–571 (433) g/d, and >571 (764) g/d for total milk; <5 (0) g/d, 5–25 (13) g/d, and >25 (40) g/d for cheese; <111 (78) g/d, 111–171 (146) g/d, and >171 (210) g/d for total meat; <96 (68) g/d, 96–155 (125) g/d, and >155 (199) g/d for red meat; <31 (13) g/d, 31–65 (45) g/d, and >65 (101) g/d for processed red meat; <48 (27) g/d, 48–87 (67) g/d, and >87 (121) g/d for unprocessed red meat; and <18 (0) g/d, 18–55 (36) g/d, and >55 (89) g/d for fish, respectively.

* $P<0.05$ and ** $P<0.01$ compared to the 1st tertile.

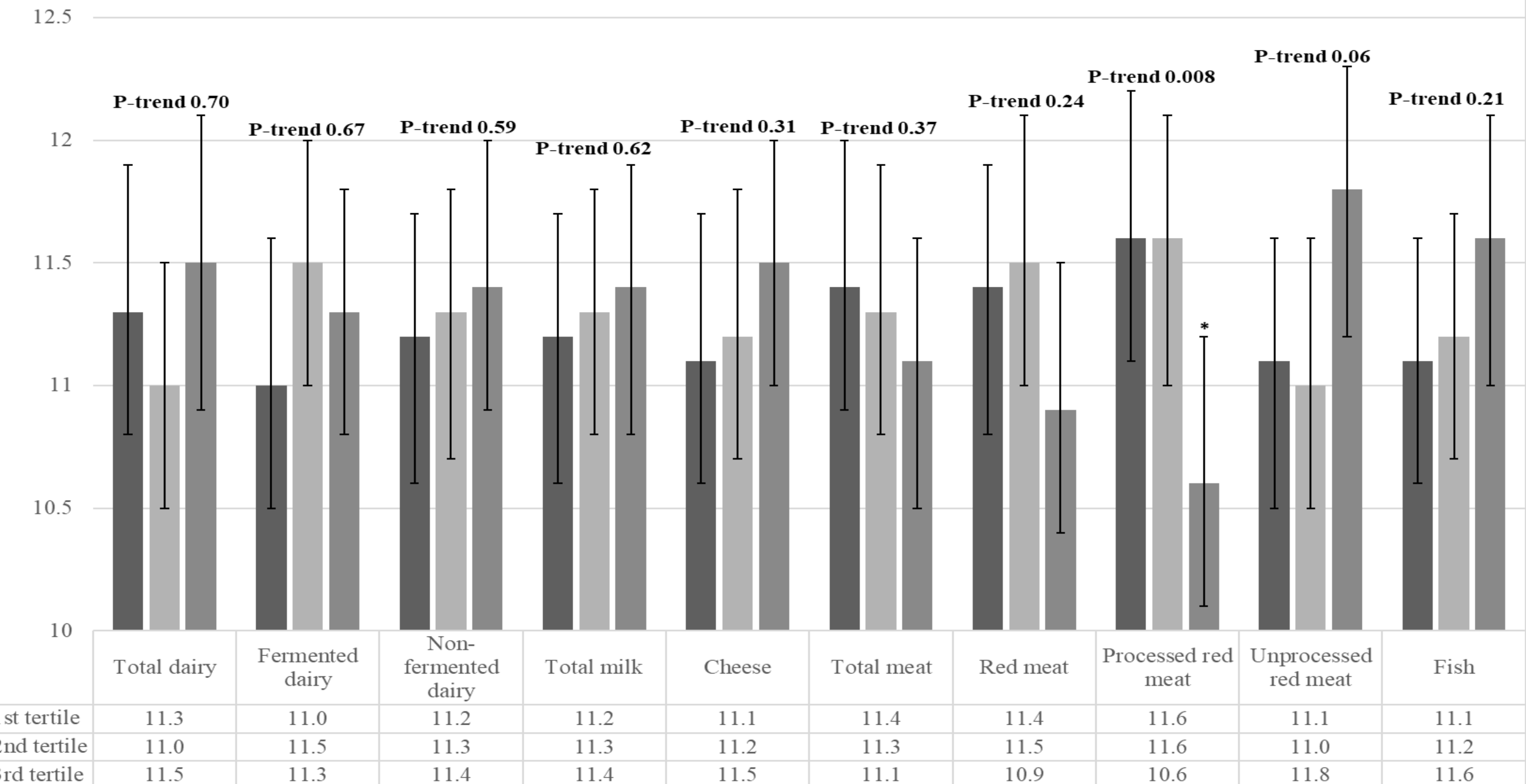

	Total dairy	Fermented dairy	Non-fermented dairy	Total milk	Cheese	Total meat	Red meat	Processed red meat	Unprocessed red meat	Fish
1st tertile	11.3	11.0	11.2	11.2	11.1	11.4	11.4	11.6	11.1	11.1
2nd tertile	11.0	11.5	11.3	11.3	11.2	11.3	11.5	11.6	11.0	11.2
3rd tertile	11.5	11.3	11.4	11.4	11.5	11.1	10.9	10.6	11.8	11.6

SUPPLEMENTAL FIGURE 11

Cognitive performance in the Russell's adaptation of the Visual Reproduction Test after 4 y of follow up in tertiles of baseline dairy, meat, and fish intakes among 482 men from the Kuopio Ischaemic Heart Disease Risk Factor Study.

The bars and the values under the figure show the mean number of correct answers (the error bars illustrating the 95% CIs). Values were obtained by ANCOVA and were adjusted for age, baseline examination year, and energy intake, education years, pack-years of smoking (cigarette packs/day × years of smoking), body mass index (kg/m^2), diabetes (yes/no), leisure-time physical activity (kcal/day), history of coronary heart disease (yes/no), use of lipid-lowering medication (yes/no), intakes of alcohol (g/week), fiber (g/day), sum of fruits, berries and vegetables (g/day), and dietary fat quality (ratio of polyunsaturated fatty acids plus monounsaturated fatty acids to saturated fatty acids plus trans fatty acids).

The intakes in the tertiles (medians in the parentheses) were <538 (353) g/d, 538–804 (663) g/d, and >804 (970) g/d for total dairy; <45 (10) g/d, 45–227 (115) g/d, and >227 (375) g/d for fermented dairy; <307 (185) g/d, 307–594 (455) g/d, and >594 (797) g/d for non-fermented dairy; <279 (165) g/d, 279–571 (433) g/d, and >571 (764) g/d for total milk; <5 (0) g/d, 5–25 (13) g/d, and >25 (40) g/d for cheese; <111 (78) g/d, 111–171 (146) g/d, and >171 (210) g/d for total meat; <96 (68) g/d, 96–155 (125) g/d, and >155 (199) g/d for red meat; <31 (13) g/d, 31–65 (45) g/d, and >65 (101) g/d for processed red meat; <48 (27) g/d, 48–87 (67) g/d, and >87 (121) g/d for unprocessed red meat; and <18 (0) g/d, 18–55 (36) g/d, and >55 (89) g/d for fish, respectively.

* $P<0.05$ compared to the 1st tertile**.**

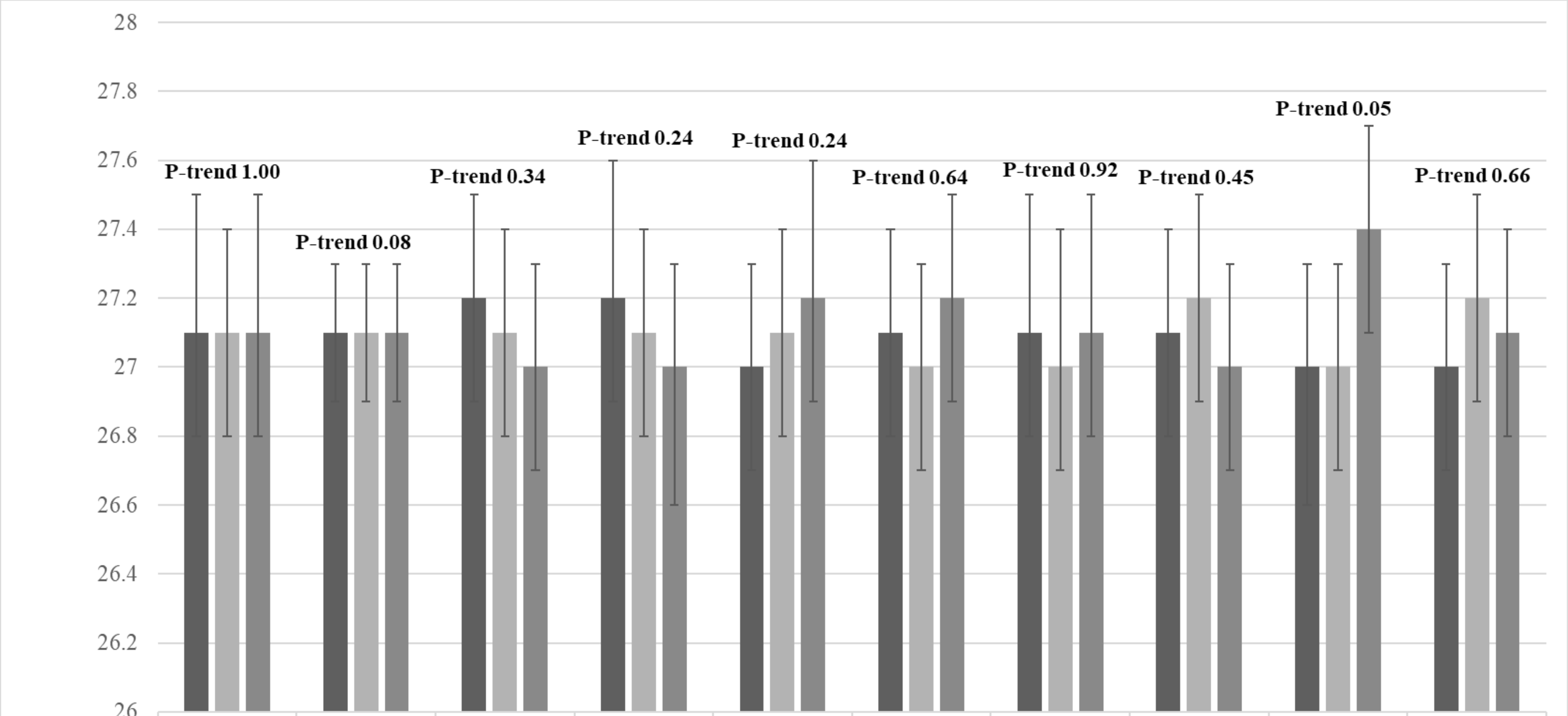

	Total dairy	Fermented dairy	Non-fermented dairy	Total milk	Cheese	Total meat	Red meat	Processed red meat	Unprocessed red meat	Fish
1st tertile	27.1	27.1	27.2	27.2	27.0	27.1	27.1	27.1	27.0	27.0
2nd tertile	27.1	27.1	27.1	27.1	27.1	27.0	27.0	27.2	27.0	27.2
3rd tertile	27.1	27.1	27.0	27.0	27.2	27.2	27.1	27.0	27.4	27.1

SUPPLEMENTAL FIGURE 12

Cognitive performance in the Mini Mental State Exam after 4 y of follow up in tertiles of baseline dairy, meat, and fish intakes among 482 men from the Kuopio Ischaemic Heart Disease Risk Factor Study.

The bars and the values under the figure show the mean score (the error bars illustrating the 95% CIs). Values were obtained by ANCOVA and were adjusted for age, baseline examination year, and energy intake, education years, pack-years of smoking (cigarette packs/day × years of smoking), body mass index (kg/m^2), diabetes (yes/no), leisure-time physical activity (kcal/day), history of coronary heart disease (yes/no), use of lipid-lowering medication (yes/no), intakes of alcohol (g/week), fiber (g/day), sum of fruits, berries and vegetables (g/day), and dietary fat quality (ratio of polyunsaturated fatty acids plus monounsaturated fatty acids to saturated fatty acids plus trans fatty acids).

The intakes in the tertiles (medians in the parentheses) were <538 (353) g/d, 538–804 (663) g/d, and >804 (970) g/d for total dairy; <45 (10) g/d, 45–227 (115) g/d, and >227 (375) g/d for fermented dairy; <307 (185) g/d, 307–594 (455) g/d, and >594 (797) g/d for non-fermented dairy; <279 (165) g/d, 279–571 (433) g/d, and >571 (764) g/d for total milk; <5 (0) g/d, 5–25 (13) g/d, and >25 (40) g/d for cheese; <111 (78) g/d, 111–171 (146) g/d, and >171 (210) g/d for total meat; <96 (68) g/d, 96–155 (125) g/d, and >155 (199) g/d for red meat; <31 (13) g/d, 31–65 (45) g/d, and >65 (101) g/d for processed red meat; <48 (27) g/d, 48–87 (67) g/d, and >87 (121) g/d for unprocessed red meat; and <18 (0) g/d, 18–55 (36) g/d, and >55 (89) g/d for fish, respectively.

There were no statistically significant differences between the groups.

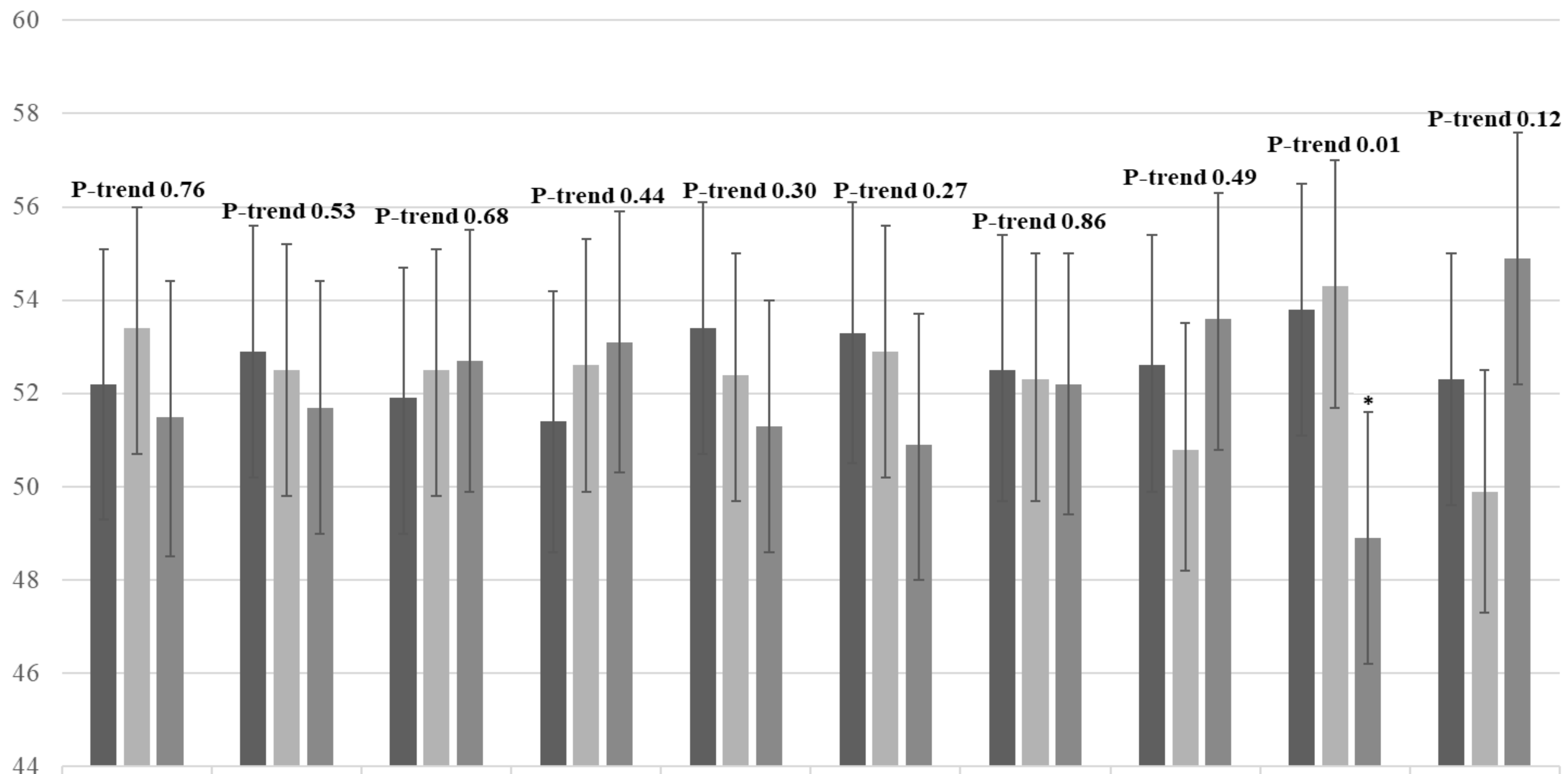

	Total dairy	Fermented dairy	Non-fermented dairy	Total milk	Cheese	Total meat	Red meat	Processed red meat	Unprocessed red meat	Fish
1st tertile	52.2	52.9	51.9	51.4	53.4	53.3	52.5	52.6	53.8	52.3
2nd tertile	53.4	52.5	52.5	52.6	52.4	52.9	52.3	50.8	54.3	49.9
3rd tertile	51.5	51.7	52.7	53.1	51.3	50.9	52.2	53.6	48.9	54.9

SUPPLEMENTAL FIGURE 13
Cognitive performance in the Trail Making Test A after 4 y of follow up in tertiles of baseline dairy, meat, and fish intakes among 482 men from the Kuopio Ischaemic Heart Disease Risk Factor Study.

The bars and the values under the figure show the mean time in seconds (the error bars illustrating the 95% CIs). Values were obtained by ANCOVA and were adjusted for age, baseline examination year, and energy intake, education years, pack-years of smoking (cigarette packs/day × years of smoking), body mass index (kg/m^2), diabetes (yes/no), leisure-time physical activity (kcal/day), history of coronary heart disease (yes/no), use of lipid-lowering medication (yes/no), intakes of alcohol (g/week), fiber (g/day), sum of fruits, berries and vegetables (g/day), and dietary fat quality (ratio of polyunsaturated fatty acids plus monounsaturated fatty acids to saturated fatty acids plus trans fatty acids).

The intakes in the tertiles (medians in the parentheses) were <538 (353) g/d, 538–804 (663) g/d, and >804 (970) g/d for total dairy; <45 (10) g/d, 45–227 (115) g/d, and >227 (375) g/d for fermented dairy; <307 (185) g/d, 307–594 (455) g/d, and >594 (797) g/d for non-fermented dairy; <279 (165) g/d, 279–571 (433) g/d, and >571 (764) g/d for total milk; <5 (0) g/d, 5–25 (13) g/d, and >25 (40) g/d for cheese; <111 (78) g/d, 111–171 (146) g/d, and >171 (210) g/d for total meat; <96 (68) g/d, 96–155 (125) g/d, and >155 (199) g/d for red meat; <31 (13) g/d, 31–65 (45) g/d, and >65 (101) g/d for processed red meat; <48 (27) g/d, 48–87 (67) g/d, and >87 (121) g/d for unprocessed red meat; and <18 (0) g/d, 18–55 (36) g/d, and >55 (89) g/d for fish, respectively.

* $P<0.05$ compared to the 1st tertile.
